# Antimicrobial Nano-Agents: The Copper Age

**DOI:** 10.1021/acsnano.0c10756

**Published:** 2021-04-01

**Authors:** Maria Laura Ermini, Valerio Voliani

**Affiliations:** Center for Nanotechnology Innovation @NEST, Istituto Italiano di Tecnologia, Piazza San Silvestro, 12-56126 Pisa, Italy

**Keywords:** copper, nanoparticles, antimicrobials, wound healing, communicable diseases, virus, bacteria, biodistribution, antiviral, ADMET

## Abstract

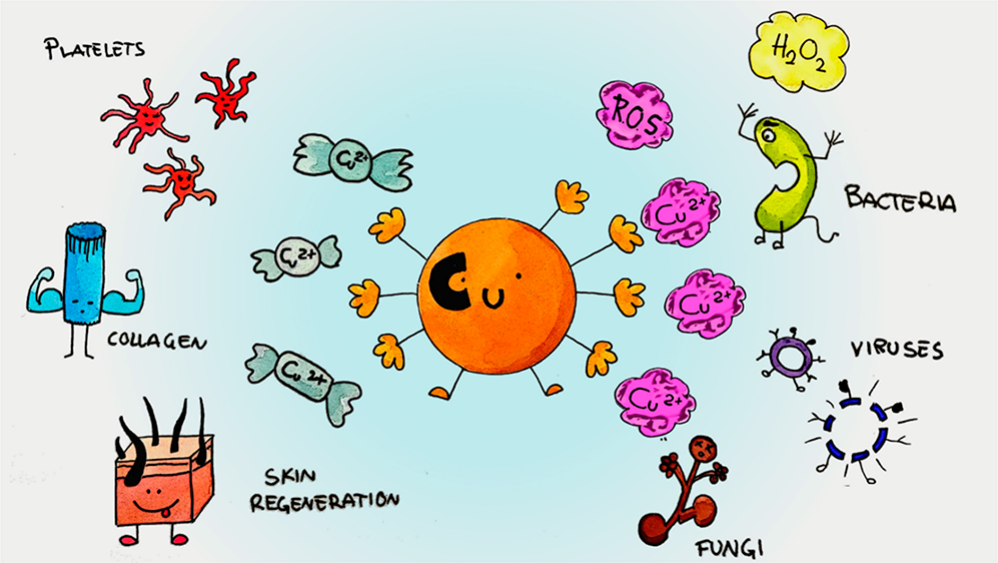

The constant advent of major health threats such as antibacterial resistance or highly communicable viruses, together with a declining antimicrobial discovery, urgently requires the exploration of innovative therapeutic approaches. Nowadays, strategies based on metal nanoparticle technology have demonstrated interesting outcomes due to their intrinsic features. In this scenario, there is an emerging and growing interest in copper-based nanoparticles (CuNPs). Indeed, in their pure metallic form, as oxides, or in combination with sulfur, CuNPs have peculiar behaviors that result in effective antimicrobial activity associated with the stimulation of essential body functions. Here, we present a critical review on the state of the art regarding the *in vitro* and *in vivo* evaluations of the antimicrobial activity of CuNPs together with absorption, distribution, metabolism, excretion, and toxicity (ADMET) assessments. Considering the potentiality of CuNPs in antimicrobial treatments, within this Review we encounter the need to summarize the behaviors of CuNPs and provide the expected perspectives on their contributions to infectious and communicable disease management.

## Introduction

Infectious diseases are one of the major global health threats. Bacteria, viruses, and other microbes still cause millions of deaths every year, despite the wide development and commercialization of antivirals and antibiotics.^1,2^

Furthermore, an increasing occurrence of multi-drug-resistant (MDR) bacteria caused by antimicrobial drug consumption has been recognized in the past decades and still holds the attention of international health organizations.^3^ In this regard, methicillin-resistant *Staphylococcus aureus* (MRSA) is one of the most dangerous resistant bacteria, being able to cause local skin infections, pneumonia, pericarditis, sepsis, and death.^3^ The World Health Organization recommendations are to limit the exposure of bacteria to antibiotics in order to avoid the evolution and spread of resistance.^4,5^ Indeed, increased awareness in the usage of existing drugs is one of the primary steps toward the reduction of the risks associated with MDR bacteria.^4,5^ On the other hand, the development of approaches for the eradication of infections from persistent bacteria can help to avoid the issue of growing multi-drug resistance.^6,7^ Among other microbes, viral infections are currently a huge concern and cause millions of deaths and hospitalizations every year. Examples include the avian influenza A (H5N1), the outbreak of severe acute respiratory syndrome (SARS), and the novel coronavirus (CoV) designated SARS-CoV. The high incidence of virus-related infections requires the discovery of effective and specific antiviral drugs and vaccines, which is very time demanding.^1^

In this scenario, the exploration of approaches for antimicrobial treatment and prevention is of special interest. In particular, the overarching goal of this field is to improve the strategies for the care of MDR-related infections by avoiding their diffusion as well as filling the lack of drugs that can target a broad spectrum of viruses.^4,8^ In an era of declining antimicrobial discovery and rapidly emerging antibacterial resistance, treatment strategies based on metal nanoparticles technology are urgently required.

The combination of the antimicrobial activity of metals with nanotechnologies is increasingly being touted as the last-line defense toward communicable diseases.^9,10^ Indeed, metal-based nanoparticles (NPs) have the peculiarity to be able to target multiple biomolecules and microbes compromising the development of resistant strains, as well as to be effective as antimicrobial agents through different mechanisms with respect to the classical treatments.^11^

The antimicrobial properties of bulk metals have been exploited for thousands of years.^12^ For example, Cu and Ag have been used for water sanitization and food preservation since the time of the Persian kings, Ag foils have been historically used to prevent infection of surgical wounds, and compounds of copper have been extensively applied in agriculture as fungistatic on grapes and potatoes.^12^ Compared to silver and gold, copper is cheaper and more easily accessible, while copper NPs (CuNPs) are biocompatible and can be synthesized by eco-friendly methods.^13^ Copper-based nanoparticles have found several applications in many areas such as additives in skin product, metal coatings, inks, and plastic for food packaging.^14^ In alloys, CuNPs have found applications in antimicrobial coatings in dentistry and virus disinfection.^15^

CuNPs demonstrate several crucial advantages with respect to other metallic NPs, among which are their cheaper and easier production.^16,17^ CuNPs dissolve faster than other noble metals by releasing ions in the surroundings.^18^ Concerning their antimicrobial activity, CuNPs generate oxidative stress, cause the disassembly of viruses or bacterial membranes, and can interfere with virus activity.^19^ At the same time, copper ions increase the generation of compounds that are toxic for microbes. Copper is an essential element for human and other mammalian life, and it is involved in several pathways that are essential for living.^20−22^ Thus, the copper ion release can enhance and support some of these pathways, such as the regeneration of tissues.^20−22^ On the other hand, CuNPs may accumulate in the body^23,24^ or release too many ions, causing long-term toxicity or contributing to the development of related diseases.^25^

In this Review, we have systematically analyzed the literature on the antimicrobial activity of copper-based NPs, critically reviewing the *in vitro* and *in vivo* antimicrobial studies for clinical purposes of pure metallic CuNPs, copper oxide nanoparticles (CuONPs), and copper sulfide nanoparticles (CuSNPs). While many works can be found about noble metal NPs, a more reduced group of works accounts for copper, and even less discusses comprehensively the potentiality of CuNPs. Nowadays, the peculiarities of CuNPs are still not completely recognized, possibly because CuNPs tend to dissolve as ions more easily with respect to other noble metals and because of the delicate equilibrium between their action and toxicity. This Review addresses this need in order to gather the behaviors of CuNPs together with their potentially relevant applications and the perspectives on their contributions to the next clinical applications in infectious and communicable diseases.

## Antimicrobial Action of Copper Nanoparticles

Even though copper is a fundamental element involved in several biological processes in living systems, it can exert a not-negligible toxic action on cells.^18,26^*In vitro* studies demonstrate that, at a certain range of concentrations, CuNPs can reduce the viability of cells, depending on the chemical composition, shape, and size of the nanomaterial. Karlsson *et al*.^26^ showed that CuONPs (10–50 nm diameter) can cause oxidative stress and DNA damage to lung epithelial cells, reducing their viability to 50% after 18 h of exposure to 20 μg/mL (for more insights about CuNPs’ toxicity, refer to Biodistribution, Toxicity, and Persistence of CuNPs). In addition to oxidative stress, CuNPs can alter the metal ion homeostasis of cells. Each cell has a complex network of internal processes that regulates the concentration of copper inside a specific range. If cells are exposed to a higher dose than the amount that can be metabolized, the system can collapse.^20^ Compared to the other metals commonly used in antimicrobial application, copper undergoes oxidation more easily and tends to dissolve in media as ions.^28,29^ Copper ions and CuNPs can be considered redox-active species.^30^ Indeed, they can subject the cell to oxidative stress through the generation of reactive oxygen species (ROS) which damage cellular components such as proteins, lipids, and nucleic acids. ROS are highly reactive species formed during the common cellular metabolism of oxygen, like ions or small molecules that contain peroxide, free radicals, or oxygen ions. They are normally involved in cell signaling, and cells are able to regulate their concentration thanks to several enzymes such as catalase or superoxide dismutase (SOD). When the ROS concentration is increased by the presence of external factors, like Cu species, the cell is subjected to oxidative stress that can bring to apoptosis.^30^

Several investigations have explored the different action that CuNPs and CuONPs exert on microbes.^31,32^ In general, CuNPs are more prone to interact with the membrane of bacteria compromising its integrity.^26^ Instead, CuONPs tend to penetrate the membrane of bacteria depending on the NP shape and releasing ions within the cell (the Trojan horse mechanism).^31,32^ When NPs are immobilized on a surface of sol–gel silica, CuNPs exhibits a stronger antibacterial activity than CuO NPs.^3^ The usual process that takes place for CuNPs is based on the redox equation which involves the energetically favorable oxidation of copper to Cu^2+^ (Cu(s) + O_2_ + 2H^+^ = Cu^2+^ + H_2_O_2_). Thus, the local production of hydrogen peroxide (and not the presence of ions) causes membrane damage.^33^ This was confirmed by the work of Cronholm *et al*. that showed a minor impact on cell membrane when exposed to Cu ionic species.^34^ Applerot *et al*. noticed that also CuONPs can produce ROS. Interestingly, *E. coli* is more affected by CuONPs than *S. aureus*, suggesting that the membrane differences between Gram-positive and Gram-negative bacteria can influence the resistance to ROS.^30^ Furthermore, they evidenced a correlation between the amount of killed bacteria and the size of the NP. Briefly, they compared CuONPs of 30 and 2 nm diameter and highlighted that smaller NPs are more effective against bacteria. Indeed, 2 nm CuONPs disrupt and penetrate the bacterial membrane more efficiently than 30 nm CuONPs, as confirmed by transmission electron microscopy (TEM) images. Furthermore, smaller NPs are associated with an increased amount of superoxide anions, generating more intense oxidative stress.^30^

Laha *et al*.^35^ demonstrated that the shape of the NP significantly influences the overall antibacterial activity by comparing the minimum inhibitory concentration (MIC) and minimum bactericidal concentration (MBC) of spherical CuONPs (33 nm) and nanosheets of CuO (257 × 42 nm). The first inhibit completely the growth of Gram-negative bacteria *Proteus vulgaris* and *E. coli* at concentrations of 0.16 and 0.20 mg/mL, while the second are more active on Gram-positive bacteria *Bacillus subtilis* and *Micrococcus luteous* (0.22 and 0.20 mg/mL, respectively). Both nanomaterials are able to damage the membrane of the bacteria (evaluated with electron microscopy) and DNA. Accordingly, with a higher dose of CUNPs (3200 μg/mL), Betancourt-Galindo *et al*. inhibited *S. aureus* and *P. aeruginosain vitro* above 99%.^36^

Zhou *et al*.^37^ prepared 800 nm Cu_2_O@ZrP hybrid nanosheets with *in situ* reduction of Cu^2+^ on the surface of ZrP. They reported that this nanomaterial is highly effective (99% after 6 h) against two superbugs: methicillin-resistant *Staphylococcus aureus* (MRSA) and vancomycin-resistant *Enterococcus* (VRE). This strong effect is associated with the depolarization and damaging of the membrane of the bacteria. Moreover, ROS are produced and cause a toxic environment. These features can be also exploited in material coatings in order to avoid microbial proliferation. In this regard, CuONPs are particularly interesting against biofilm formation.^38^ Zn-doped CuONPs have been deposited on catheters *via* ultrasound nanofabrication and employed against three pathogens commonly found in the urinary tract (*E. coli*, *S. aureus*, and *Proteus mirabilis*).^38^ These substrates, tested *in vivo* on rats, have been able to delay or avoid urinary infection until day 7.

Several of the CuNPs evaluated for their antibacterial activity (Table 1) are biosynthesized. Natural compounds, directly extracted from plants or fermented, are often used as stabilizers for CuNPs synthesis. In this regard, plant-extracted compounds are generally ecofriendly and biocompatible, have low-toxicity, and may synergistically enhance the antimicrobial performances of CuNPs.^39^ We would like to stress that the focus of this Review is to comprehensively discuss the antimicrobial activity of CuNPs. Thus, in the following we are just reporting a selection of the syntheses proposed to produce CuNPs. For a discussion with a focus on the production, we suggest the readers look elsewhere.^40−42^

**Table 1:** Summary of Size, Shape (Sphere-like if Not Specified), and Application of CuNPs

authors/ref	NP size	target bacteria	application *in vitro*
Betancourt-Galindo *et al*., 2014^36^	4–12 nm	*Pseudomonas aeruginosa* (ATCC 13388) and *Staphylococcus aureus* (ATCC 6538)	in Petri dish
Rasool and Hemalatha, 2017^43^	–	*Klebsiella pneumoniae*, *Proteus mirabilis*, *Escherichia coli*, *Salmonella typhimurium*, and methicillin-resistant *S. aureus*	in Petri dish
Bocarando-Chacón *et al*., 2020^44^	10 nm	*E. coli*	in Petri dish
Laha *et al*., 2014^35^	33 nm spherical,	*Bacillus subtilis* (ATCC 6633), *Micrococcus luteous* (ATCC 9341), *E. coli* (ATCC 10,536), *Proteus vulgaris* (ATCC 13,387), and DH5a (kl2)	in Petri dish
	257 × 42 nm sheet		
Chowdhury *et al*., 2013^81^	3 nm	*S. aureus* and *E. coli*	in natural fibers, oil palm empty fruit bunch fiber
Roy *et al*., 2016^13^	15 nm	*S. aureus*, *Pseudomonas putida*, and *E. coli*	using the leaf extract of *Meliconia psittacorum*
Marković *et al*., 2020^85^	30–40 nm	Gram-negative bacteria *E. coli* (ATCC 25922), *E. coli* (ATCC BAA 2469), and *K. pneumoniae* (ATCC BAA 2146); Gram-positive bacteria *S. aureus* (ATCC 25923) and *S. aureus* (ATCC 43300); and yeast *Candida albicans* (ATCC 24433)	NPs grown on bleached cotton woven fabric
Sathiyavimal *et al*., 2018^84^	50 nm	Gram-negative (*E. coli* and *P. vulgaris*) and Gram-positive (*S. aureus*) bacteria	synthesized using *Sida acuta* leaf extract, incorporated into cotton fabric
Amorim *et al*., 2019^45^	10 nm	*S. aureus* (ATCC 29213)	cashew gum
Valencia *et al*., 2020^82^	20	Gram-negative (*E. coli*) and Gram-positive (*Listeria innocua*) bacteria	cellulose nanofibril
Shahidi *et al*., 2018^83^	40 and 100 nm	*S. aureus* (Gram-positive)	cotton fabric obtained with laser ablation
Delgado *et al*., 2011^4300^	10–40 nm	*E. coli*	in a polypropylene matrix
Yaqub *et al*., 2020^47^	20 nm	*P. aeruginosa* and *E. coli*	with doxycycline
Villanueva *et al*., 2016^75^	63–160 nm	Gram-positive (*S. aureus*) and Gram-negative (*E. coli*) bacteria	starch hydrogel
Cady *et al*., 2011^77^	–	*Acinetobacter baumannii*	on cellulose
Vuković *et al*., 2015^74^	–	*E. coli*, *S. aureus*, and *C. albicans*	hydrogel
Tang *et al*., 2018^70^	50 nm	*E. coli*	polyethylenimine-stabilized NPs, embedded in agar
El-Batal *et al*., 2018^39^	35 nm	*K. pneumoniae*, *S. aureus*, and *C. albicans*	in Petri dish
Sankaref *et al*., 2015^51^	577 nm	*K. pneumoniae*, *Shigella dysenteriae*, *S. aureus*, *Salmonella typhimurium*, and *E. coli*	copper oxide with *Ficus religiosa* leaf extract
Applerot *et al*., 2012^30^	2 and 30 nm	Gram-positive (*S. aureus*) and Gram-negative (*E. coli*) bacteria	antibacterial mechanism of CuO NPs
Balcucho *et al*., 2020^69^	191 nm	methicillin-resistant *S. aureus*	polymer (polycaprolactone)
Jayaramudu *et al*., 2020^72^	8 nm	Gram-positive (*S. aureus*) and Gram-negative (*E. coli*) bacteria	chitosan hydrogel
Gutierrez *et al*., 2019^80^	20–50 nm	*E. coli* and *S. aureus*	3D-printed alginate hydrogel
Yin *et al*., 2014^93^	40 nm	Gram-positive oxacillin drug-resistant *S. aureus* and Gram-negative kanamycin drug-resistant *E. coli*	CuS NPs on the surface of NaYF4:Mn/Yb/Er@photosensitizer-doped SiO_2_
Zhou *et al*., 2019^37^	800 nm	methicillin-resistant *S. aureus* (MRSA) and vancomycin-resistant enterococcus (VRE)	Cu_2_O@ZrP nanosheet


authors/ref	NP size	target microbes	application *in vivo*
Sankar *et al*., 2015^51^	577 nm	*K. pneumoniae*, *Shigella dysenteriae*, *S. aureus*, *Salmonella typhimurium*, and *E. coli*	wound healing on albino rats
Li *et al*., 2016^68^	40–50 nm	cell attachment and proliferation of human umbilical vein endothelial cells (HUVECs), angiogenesis-related gene expression, vascularization by ELISA	wound healing on albino rats
Zangeneh *et al*., 2019^52^	–	*Candida albicans*, *Candida glabrata*, *Candida krusei*, *Candida guilliermondii*, *P. aeruginosa*, *E. coli*, *B. subtilis*, *S. aureus*, *Salmonella typhimurium*, and *Streptococcus pneumonia*	wound healing and antioxidant
Alizadeh *et al*., 2019^50^	20, 40, 80 nm	cell migration, proliferation of endothelial and fibroblast cells, and collagen deposition	different sizes and different concentrations
Zhao *et al*., 2020^46^	30 nm	cytotoxicity, and antifungal and antibacterial screening	wound healing evaluated by the number of fibrocytes and the concentrations of hydroxyproline, hexuronic acid, and hexosamine
Zhou *et al*., 2020^90^	35 nm	–	hydrogel + photothermal
Tahvilian *et al*., 2019^63^	50 nm	four fungal species, namely *Candida albicans* (PFCC No. 89-1000), *Candida glabrata* (PFCC No. 164-665), *Candida krusei* (PFCC No. 52951), and *Candida guilliermondii*, and four bacterial species, namely *P. aeruginosa* (ATCC No. 27853), *E. coli* 0157:H7 (ATCC No. 25922), *B. subtilis* (ATCC No. 6633), *S. aureus* (ATCC No. 25923), *Salmonella typhimurium* (ATCC No. 14028), and *Streptococcus pneumonia* (ATCC No. 49619)	*Allium saralicum*, on wound healing
Gopal *et al*., 2014^53^	50 nm	–	wound healing *in vivo*, chitosan
Xiao *et al*., 2018^4301^	30 nm	drug-resistant Gram-positive *S. aureus* and Gram-negative *E. coli*	infected wound healing *in vivo*, catalytic activity with H_2_O_2_
Wang *et al*., 2020^11^	5 nm (roughness)	MRSA	in MRSA-infected wounds with GO
Qiao *et al*., 2019^91^	6 nm	drug-resistant Gram-negative bacterial ESBL *E. coli* and MRSA	in MRSA-infected wounds, photothermal with quantum dots of CuS
Tao *et al*., 2019^54^	88 nm	Gram-negative (*E. coli*) and Gram-positive (*S. aureus*)	CuNPs + hydrogel net for photothermal *in vivo* infected wounds
Shalom *et al*., 2017	35–95 nm	*E. coli*, *S. aureus*, and *Proteus mirabilis*	catheter-associated urinary tract infections prevention

For example, CuNPs synthesized in the presence of actinomycetes were more effective *in vitro* against several pathogenic bacteria (*Klebsiella pneumoniae*, *Escherichia coli*, *Proteus mirabilis*, *Salmonella typhimurium*, and MRSA) with respect to gentamicin.^43^ CuNPs produced in the presence of extracts of *Opuntia ficusindica* and *Geranium* demonstrated an antimicrobial effect on *E. coli* at a lowest concentration of NPs of 250 μg/mL.^44^ Similarly, *in vitro* inhibition of bacteria growth has been observed with CuNPs prepared in the presence of *Heliconia psittacorum*(13) or fermented fenugreek (*Trigonella foenum*) powder under the action of *Pleurotus ostreatus* or stabilized with cashew gum.^39,45^ The last work compares the action of copper ions released by copper sulfate in alginate matrix and CuNPs with ciprofloaxacin in a “zone of inhibition” test. Interestingly, the copper ion sample does not create any growth inhibition against *K. pneumoniae*, *S. aureus*, and *Candida albicans*. On the contrary, CuNPs evidenced a strong antibacterial and antifungal activity. Zhao *et al*.^46^ investigated the sensitivity of fungal and bacterial strains to CuNPs prepared with leaf extract of *Allium eriophyllum*, in comparison with the extract alone and copper sulfate alone. All tested microbes were sensitive to CuNPs and to the extract. Noticeably, in almost all cases the MIC for CuNPs was about 2 times lower than with the extract and 4 times lower than with copper sulfate.^46^ Yaqub *et al*. used extracts of ginger (*Zingiber officinale*) and garlic (*Allium sativum*) for the synthesis of CuNPs and NPs conjugated with doxycycline, an antibiotic. The antimicrobial activity of the green-synthesized NPs conjugated with the drug showed more pronounced results against *P. aeruginosa* with respect to garlic and ginger NPs, although similar to those with the drug alone.^47^

## Mechanism of Action of CuNPs on Wound Healing

The correct healing of wounds is still a challenging clinical problem frequently causing morbidity and mortality.^48^ Bacteria can easily infect the exposed tissues in the injury and consequently alter or inhibit the healing process. Sometimes the healing can stop, and the wound converts into a chronic disease with associated difficulties for its management. A plethora of nanomaterials have been developed and studied for improving the offer of medicaments for these injuries. Nano-objects, such as quantum dots, nanotubes, liposomes, and metal NPs, have been exploited for their flexible applicability, antimicrobial properties, and ability for controlled release of a cargo.^49^ Some materials are able to combine these two latter aspects, contributing at the same time to the disinfection of the wound and to the stimulation of the process of healing. In this regard, CuNPs ranging from 6 nm to about 600 nm (Table 1) can trigger ROS production, killing bacteria in the wound as well as releasing copper ions that can spur the healing process.^46,50−54^

Wound-healing evolution can be summarized in a series of cascade effects that take place after the injury occurs.^49,55^ First, the platelets of the blood stick to the exposed area of the injury in order to block the bleeding (homeostasis). They change shape for better clotting and send chemical signals to promote the process of activation of fibrin. This forms a mesh that fixes the platelets to each other. Platelets also release several pro-inflammatory factors (prostaglandins, thromboxane, prostacyclins, and histamine). Second, in the inflammation phase, the dead cells and bacteria are cleared out from white blood cells. Third, the cells migrate and divide, stimulated by the platelet-derived growth factor (PDGF).^20^ This is the proliferation phase, when the wound contracts because of tissue reconstruction. Fibroblasts form an extracellular matrix of fibronectin and collagen and contract to stretch the wound edges. In addition, blood vessels are formed (angiogenesis) and epithelialization occurs. Fibroblasts and endothelial cells are recruited from transforming growth factor beta (TGF-β) and vascular endothelial growth factor (VEGF) secreted by platelets from the first step. Finally, in the last phase of remodeling, collagen realigns and the useless cells undergo apoptosis.^46^

**Figure 1:**
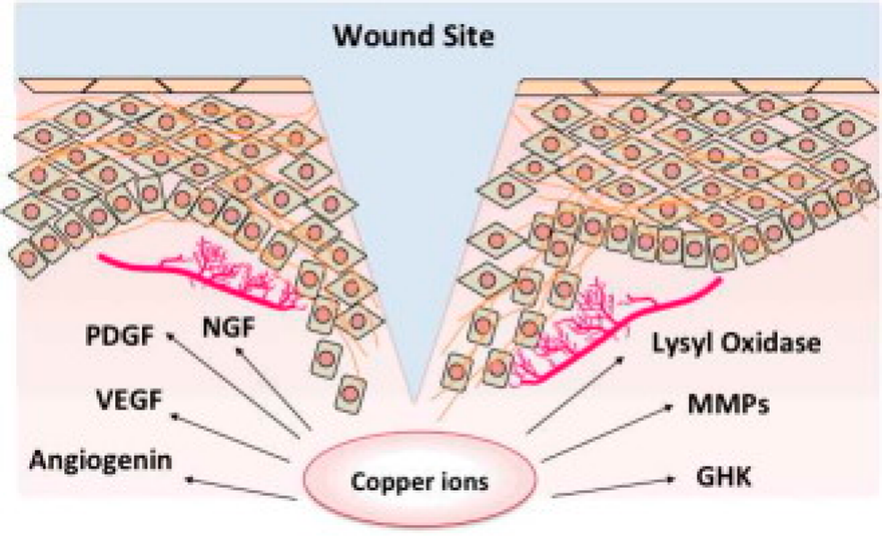
Scheme of the several reactions in which copper plays a role in wound healing. Reprinted with permission from ref (20). Copyright 2016 Elsevier.

Several reactions in which copper plays a role in wound healing are shown in Figure 1.

Copper is involved in the homeostasis phase since the cells need to internalize copper ions for its employment in several pathways naturally essential for the correct metabolism of cells. Under physiological conditions, copper is involved in several oxidation–reduction cycles thanks to the relatively easy interconversion among the different oxidation states.^56^ For instance, copper is the metal ion in cytochrome *c* oxidase involved in the biosynthesis of neurotransmitters in neuropeptide biosynthesis, or in the action of Zn-SOD against ROS.^56^ On the cell membrane, the so-called high-affinity copper uptake protein 1 (Ctr1) is responsible for copper transport inside the cell. Ions are either reduced by plasma membrane reductase or moved by copper chaperone proteins. Mitochondria can receive copper ions in order to insert them in cytochrome *c* oxidase or to redirect them to post Golgi compartments or to cytosolic enzymes. The cell is equipped with a complex system for internal regulation of the amount of copper ion, so that, when the concentration is too high, copper is moved from adenosine triphosphatase 7A (ATP7A) from the Golgi network to the membrane for expulsion.^20^

In the wound-healing process, during the proliferation phase, PDGF-induced migration of vascular smooth muscle cells (VSMCs) is dependent on ATP7A activity, which is copper dependent. In the presence of a wound scratch, if ATP7A is depleted, VSMC migration is inhibited and the decrease of copper concentration is blocked, too.^57^ In the same phase and in the following one, i.e., proliferation and remodeling, copper also acts in a complex with a peptide (GHK, glycyl-l-histidyl-l-lysine) to enhance the synthesis of collagen and to modulate the activity of metalloproteinases.^58^ At the end of proliferation phase, the wounded skin becomes stronger thanks to aldehyde cross-links on collagen and elastin. This process is catalyzed by the enzyme lysyl oxidase, which is copper dependent: low concentration of copper leads to poor wound healing.^20^

Copper availability is also implicated in angiogenesis: the stimulation of vessel formation is imputed to the copper regulation of angiogenin and VEGF, the most effective mediator in the process of vessel formation. In particular, the concentration of copper ion can accelerate the wound healing and favors the creation of a tissue with better quality (high density of the cells’ hyperproliferative epidermis), as demonstrated by Sen *et al*. by treating wounds in mice with copper sulfate.^59^ Nerve growth factor (NGF) contributes to angiogenesis, too, and its function is modulated by copper concentration. Copper can trigger NGF for neurite outgrowth or can inhibit NGF-mediated survival, avoiding the cell death induced by hydrogen peroxide from oxidative stress.^60^

As they are involved in so many fundamental processes, CuNPs can provide the copper needed for basic functionalities as well as supplement the system with a boost of metal, speeding up and promoting the quality of the overall process.

## CuNPs as Antimicrobial and Wound-Healing Enhancers

The wound-healing potential of CuNPs has been recently subjected to several investigations. The antimicrobial activity is synergic with the healing effect since the presence of infection can enhance and prolong inflammation and slow down the healing process. Zhao *et al*.^46^ demonstrated that CuNPs prepared with *A. eriophyllum* leaf extract can remove bacteria and fungi in wounds while contributing to the healing of cutaneous injuries. Their NPs were able to increase the fibrocyte/fibroblast ratio at day 10 from administration compared with samples treated with copper ions or plant extract alone. Furthermore, they observed a regulation of the inflammation (lymphocytes, macrophages, and neutrophils).^46^ Copper oxide NPs on wounds in male Wistar Albino rats prevented bleeding, microbial infection, and pus formation from the first days of treatment (NPs daily applied topically, for a period of 12 days, 20 mg/kg body weight), differently from control groups.^51^ The wound size was more reduced in treated wounds compared with untreated animals, starting from the fourth day. Wounds treated with CuONPs showed higher degrees of re-epithelialization, higher amounts of collagen, and improved formation of capillaries.

Zangeneh *et al*.^52^ demonstrated *in vitro* the antibacterial and antifungal properties of green-synthesized CuNPs from an aqueous extract of *Falcaria vulgaris* on a wide group of microbes with Agar well-diffusion and disk-diffusion methods (see Table 1). Akin to the paper described above, they confirmed that the wound-healing improvement resulted from the application of CuNPs on wounds *in vivo*. In particular, they observed an increase of fibrocytes, hexosamine, hexuronic acid, and hydroxyproline (major components of collagen). Furthermore, they noticed a regulation in the number of lymphocytes, macrophages, and neutrophils at day 10, meaning that the inflammation process is regulated and the healing is facilitated.^52^ Tahvilian *et al*.^63^ obtained comparable results with CuNPs prepared with *Allium saralicum*. The antibacterial and antifungal activities were compared with those of copper sulfate alone, the plant extract alone, and some common antibiotics and antifungal compounds. Almost all of the tested bacteria and fungi were affected by CuNPs@Allium, and their antibacterial and antifungal activities were more intense than those of standard antibiotics. The wound-healing effect was comparable with that reported in the paper from Zangeneh *et al*., suggesting that the antioxidant activity of CuNPs, measured by 2,2-diphenyl-1-picrylhydrazyl (DPPH) assay, also contributes to moderate the inflammation and the pus production in the wounds.

Alizadeh *et al*. accounted for the influence of size and concentration on antimicrobial activity of CuNPs in the *in vivo* wound treatment. Several concentrations (1 μM, 10 μM, 100 μM, 1 mM, and 10 mM) and three NP diameters (20, 40, and 80 nm) were considered. The overall results did not indicate any defined trend. However, the smaller NPs were cytotoxic at all concentrations for endothelial cells, while they had no effect on viability of fibroblast and keratinocyte cells. The bigger NPs at the lower concentrations did not show accumulation in liver of the mice, which they enhanced endothelial cell migration and proliferation and stimulated tissue granulation and the growth of blood vessels. Similar results were obtained for 40 nm NPs and 1 mM concentration.^50^

CuNPs combined with other NPs were recently described as a highly effective antimicrobial wound healers both *in vitro* and *in vivo*.^11^ In particular, CuS NPs were integrated with graphene oxide (GO) nanosheets to compose nanomaterials (CuS/GO nanocomposites (NCs)) that can both physically and chemically act on bacteria. In CuS/GO NCs, graphene nanosheets have a destructive effect on bacteria by two mechanisms: (i) the cell membrane of bacteria is damaged by severe insertion and cut, and (ii) lipid molecules are directly extracted, causing the collapse of the structure.^64^ Briefly, this nanocomposite has a peculiar morphology that destroys bacteria through the “blade effect” (wrapping the cellular membranes) and damages the cell membrane by the needle-like structure. Furthermore, CuS/GO NCs have a peroxidase activity, generate ROS, favor the cell migration, and demonstrate low cytotoxicity and biocompatibility. CuS/GO NCs totally inhibit *in vitro* both *E. coli* and MRSA at a concentration of 100 μg/mL. Bacterial live/dead staining demonstrates the efficient killing property of CuS/GO NCs. After 24 h of contact, almost all bacteria were killed *in vitro*, in contrast to the use of GO alone, which killed only the half of the population in the same conditions. Both CuS/GO NCs and GO induce changes in bacteria morphology *via* cytoplasm shrinking and damage to the membrane. In addition, the adhesion of the bacteria is drastically reduced. This nanocomposite accelerates the wound healing *in vivo* on MRSA-infected wounds and eliminates the bacteria after 14 day of treatment.

In summary, these experimental works confirm the ability of CuNPs to assist and increase the wound-healing process and synergistically sanitize the surroundings with a combination of effects that depends on the size, the chemical nature, and the design of the nanomaterial.

## CuNPs Embedded in a Matrix: Wound Dressing

The risk that a wound can be infected is reduced if the wound site is covered with a material with specific antimicrobial properties. The suitable wound dressing should first create a layer on the exposed tissues in order to provide sterile protection against external pathogens and further traumas. It should be biocompatible so as to not affect the viability of the cells, and it should keep the conditions for better healing (i.e., keep the wound site moist to enhance the cell migration). Polymers, hydrogels, creams, tissues, and fibers are just some of the materials proposed in the literature as suitable candidates in wound healing.^55^ Overall, infections can be prevented while promoting healing by incorporating an active agent inside the material. NPs can find a useful application in this scenario,^65,66^ and CuNPs as antimicrobial agents and wound-healing promoters are increasingly incorporated in membranes, polymers, polysaccharides, hydrogels, fibers, and tissues, as discussed below. It is worth noting that such topical pharmaceuticals, like the copper-based ones, constitute one of the last resources for wound healing and are usually effectively incorporated with other therapeutics for enhancing the natural healing process and considering the overall clinical condition of the patient.^67^

### Membrane

**Figure 2:**
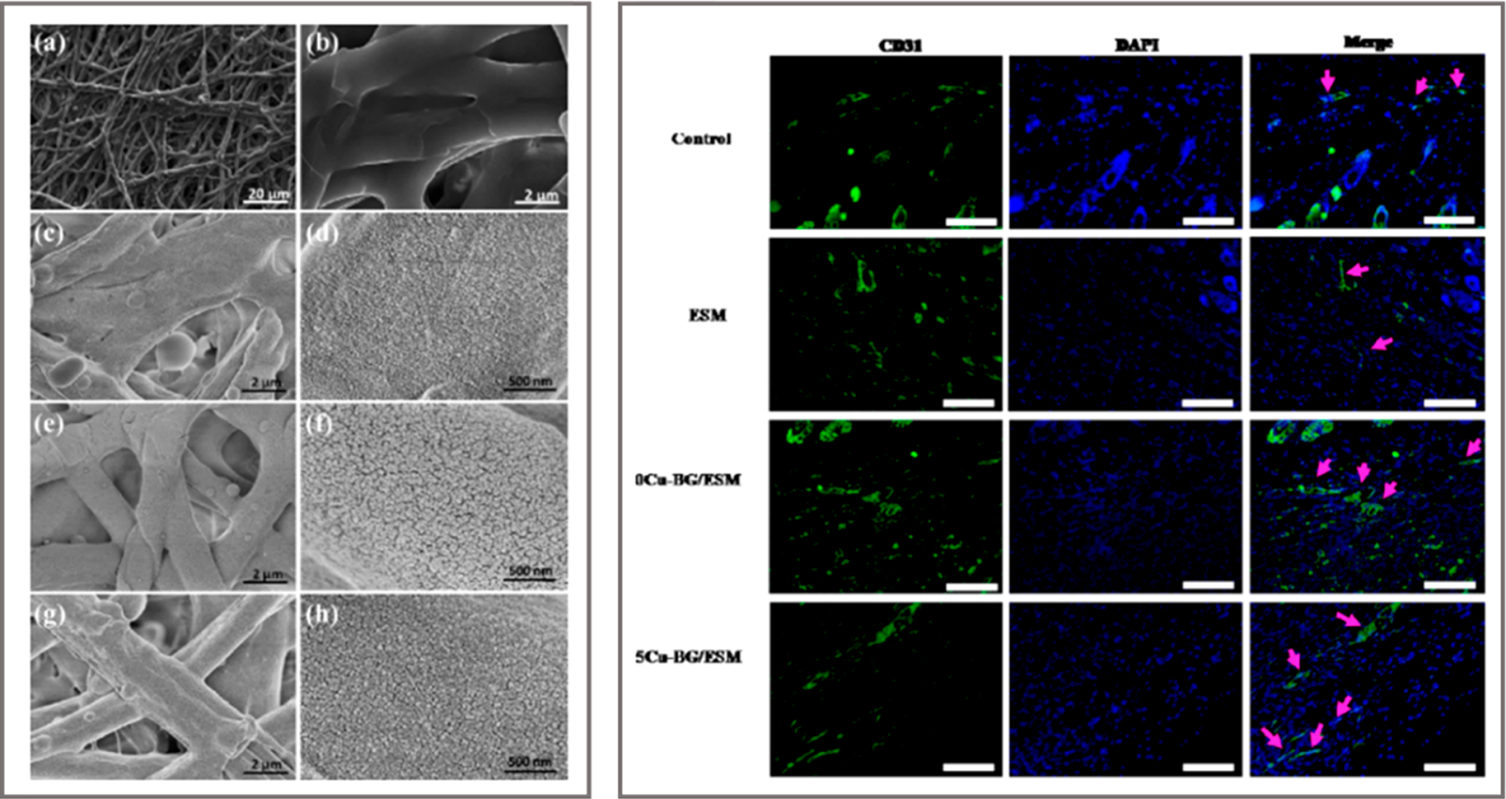
Left: SEM images showing the morphology of substrates with different amount of CuNPs at two magnifications: (a, b) outer eggshell membrane (ESM), (c, d) 0Cu-BG/ESM, (e, f) 2Cu-BG/ESM, and (g, h) 5Cu-BG/ESM. Right: Detection of increased vessel by immunofluorescence of CD31 (green) at day 7. Nuclei are stained with DAPI (blue). Vascularized areas are indicated by pink arrows. Scale bar = 100 μm. Reprinted with permission from ref (68). Copyright 2016 Elsevier.

A very particular substrate for wound dressing was reported by Li *et al*.^68^ They combined CuNPs-coated bioactive glass (Cu-BG) with natural eggshell membrane (ESM). The ESM is peeled off from eggs, dried, and subsequently decorated with Cu-containing glass ceramic disks *via* laser ablation. The purpose of the ESM is to improve hydrophilicity to the systems, while the decoration with copper enhances the hardness of the natural membrane. This material reduces the viability of *E. coli* of 90% both *in vitro* and *in vivo* and stimulates angiogenesis. VEGF, angiogenesis-related gene expression (VEGF, HIF-1a, VEGF receptor 2), and endothelial nitric oxide were improved *in vitro* when the dressing was tested on human umbilical vein endothelial cells (HUVECs). When applied directly on rat injuries, the substrate contributed to enhance the healing quality and time (i.e., improved angiogenesis and formation of uniform epidermis). Immunofluorescence staining of CD31, a transmembrane protein expressed early in vascular development, showed a higher density of vessels on the copper-containing substrate (5Cu-BG/ESM in Figure 2) with respect to control, ESM alone, and substrates without copper (0Cu-BG/ESM groups in Figure 2).

### Polymers and Polysaccharides

A biocompatible wound dressing for MRSA in diabetic foot ulcers was recently presented by Balcucho *et al*.^69^ They combined polycaprolactone and copper oxide NPs (191 nm) to obtain an active film able to totally inhibit MRSA in 24 h. Polycaprolactone flakes, dissolved in butanol and chloroform, were mixed with a powder of CuONPs and subsequently dried in a Petri dish. The polymer is used as an immobilization matrix for CuONPs and as a substrate for biocompatibility and antimicrobial activity tests. The material is FDA approved for therapeutic agents release, hemo-compatible (red blood cell breakage less than 5%), and stable to thermal stress, but as a side effect the fibroblast activity results *in vitro* were reduced of 20%. Tang *et al*.^70^ demonstrated that CuNPs can be stabilized as a powder for several months in air if prepared using a biocompatible polyelectrolyte: polyethylenimine (PEI). PEI-stabilized CuNPs were embedded in agar and deposited as a film. The substrate (2 × 1 cm) incubated with a bacterial suspension (*E. coli*, 2 mL, 9.0 × 10^8^ CFU/mL) demonstrated high bacterial killing efficiency for 12 h with nearly no colony-forming units (CFU) found.

**Figure 3:**
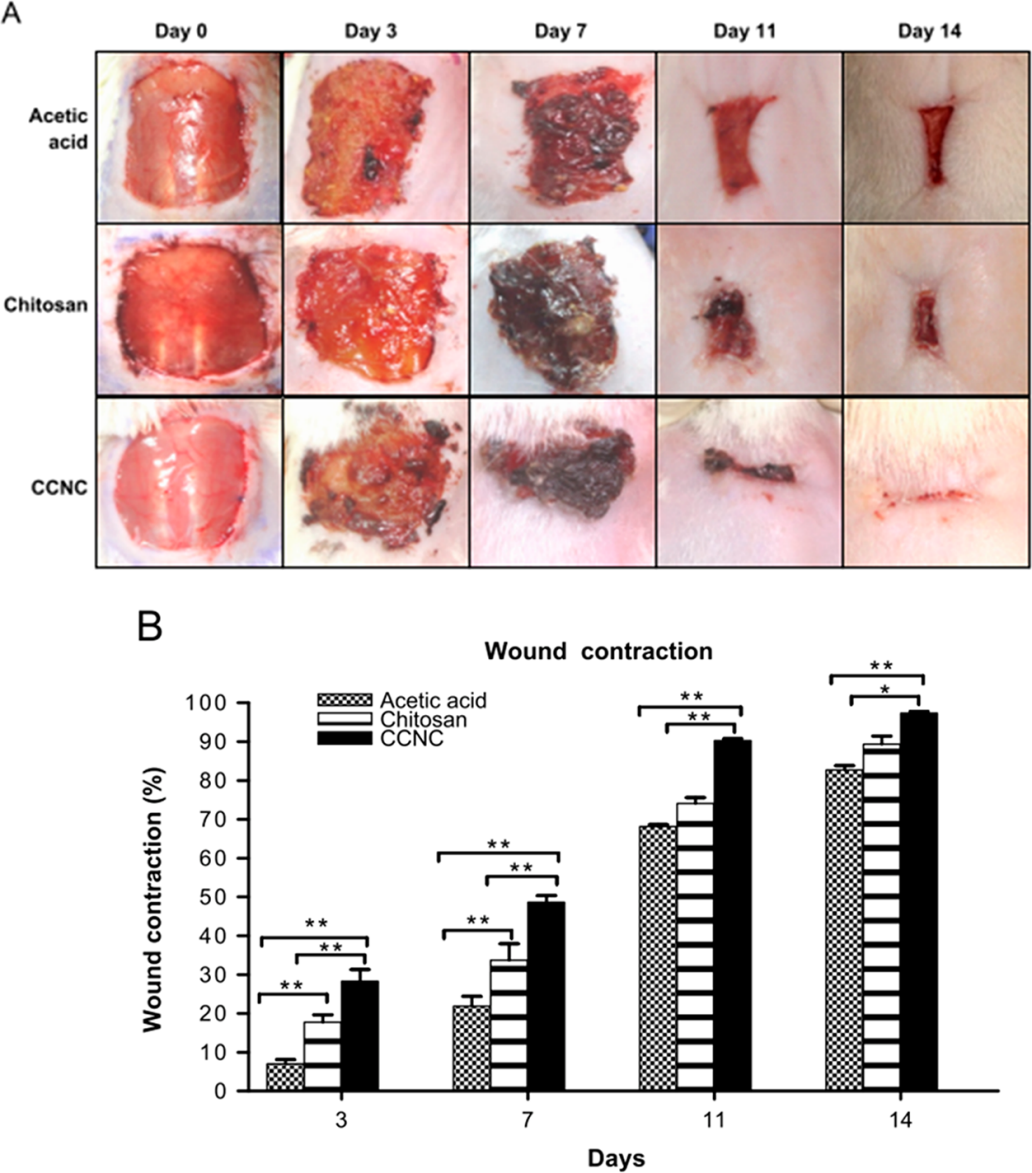
(A) Photographs of wounds at days 0, 3, 7, 11, and 14 after treatment with acetic acid, chitosan, and CCNC. (B) Wound contraction (%) at different days.^53^ Reprinted with permission from ref (53). Copyright 2014 Elsevier.

Chitosan is a linear polysaccharide known for its biocompatibility, easy biodegradability, and antimicrobial activity. When chitosan is combined with CuNPs (CCNC), the properties of the building blocks can be synergistically summed, and the resulting material will have improved ability in wound healing.^53^ In rats, CCNC composites generate relevant wound contraction and fast hair coat regeneration. CCNC is composed of a mixture of colloidal chitosan solution (10%) and 50 nm CuNPs (0.3%). As shown in Figure 3, the wound contraction % *in vivo* is higher for CCNC than for chitosan alone starting from day 3 until day 14.

As expected, VEGF is found to be higher in CCNC samples than in chitosan alone, meaning a more efficient angiogenesis. The inflammation markers such as TGF-b1 and IL-10 initially increase for monocytes and macrophages because of the inflammatory activity of the chitosan. Later, the pro-inflammatory cytokines are lower if CCNC is applied with respect to chitosan alone, since the copper attenuates the inflammation process.^65^

The effective antimicrobial activity of CCNC was demonstrated *in vitro* by Jayaramudu *et al*.^72^ against Gram-positive (*S. aureus*) and Gram-negative (*E. coli*) bacteria. The inhibition zone test showed that the concentration of copper determined the effectiveness of the substrate (800–400 μg/mL).

### Hydrogel Copper Nanocomposites

Hydrogels are three-dimensional polymer nets characterized by their abilities to imbibe aqueous solutions (water absorption capacity up to 10 g/g) and to be arranged in different shapes such as patches, gels, ointments, and films. Hydrogels can encapsulate nanomaterials, drugs, or active biomolecules and trigger on-site controlled release. They are usually biocompatible and suitable for a wide range of medical applications, among which are the smart design of engineered tissues, wound tissues, medicament patches, and contact lenses.^73^

**Figure 4:**
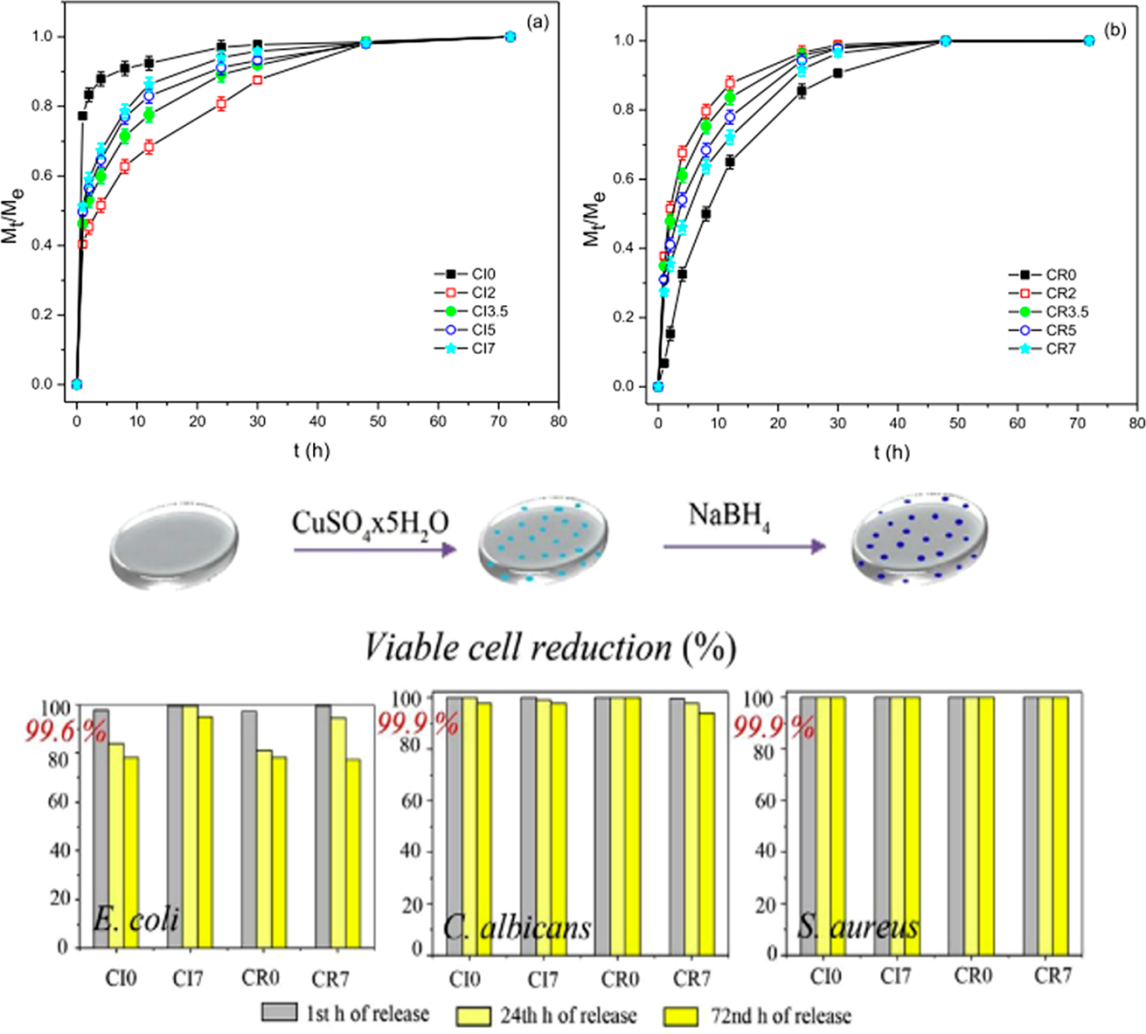
Upper graphs: Ratio between the weight of swollen hydrogel at time *t* and the weight of swollen hydrogel at equilibrium state. Lower graphs: Antibacterial activity of the hydrogels against *E. coli*, *C. albicans*, and *S. aureus* (itaconic acid concentration is varied). Reprinted with permission from ref (74). Copyright 2015 Elsevier.

Vukovic *et al*.^74^ prepared a hydrogel based on 2-hydroxyethyl acrylate and different concentrations of itaconic acid. The dried samples, immersed in a Cu^2+^ solution for 24 h, loaded the ions inside the gel. A following reduction induced the formation of CuNPs. The release profile and the antimicrobial activity are shown for incorporated copper(II) ions (CIx) and for reduced copper substrates (CRx) in Figure 4.

*M*_t_/*M*_e_ is the fractional ion release, reported in Figure 4 versus time. The loaded substrates rapidly release the copper ions in the first hour—more rapidly with increasing concentration of itaconic acid. The CuNPs substrates, in contrast, exhibit a slower release profile and with the opposite trend: as the itaconic acid content increases, the release rate decreases. The authors suggest that the increased density of the substrate influences the slower release from the reduced substrate. The antimicrobial activity has been tested *in vitro* on *E. coli*, *S. aureus*, and *C. albicans*, highlighting a reduction in bacteria and fungi population starting from the second hour, and almost completely after 24 h.

Villanueva *et al*.^75^ combined a starch hydrogel with CuNPs coated with silica. The silica was shown to improve the stability of the NPs, avoiding the fast release oxidation of metallic copper to the blue ion Cu^2+^. They were not able to detect the amount of ion released after 24 h, claiming it was too low. Different doses of NPs per grams of gel were tested for antimicrobial activity against both Gram-positive and Gram-negative bacteria *via* an inhibition zone test. The antibacterial effect was recorded for all concentrations tested, and specifically it increased with the NPs concentration (0.36–1.27 mmol of Cu per gram of gel).

The hydrogel prepared by Qui *et al*.^76^ showed peroxidase-like activity; i.e., it could convert hydrogen peroxide into ROS, which are toxic for bacteria. The hydrogel was obtained from Cu(NO_3_)_2_ and l-aspartic acids (l-Asp), resulting in a network of fibers with nanometric diameter (50–70 nm). The peroxidase activity was demonstrated by observing the oxidation of 3,3′,5,5′-tetramethylbenzidine (TMB) caused by hydrogen peroxide and with a terephthalic acid assay. The acid captures radicals from the oxidation reaction and generates fluorescent hydroxyterephthalic acid (TAOH). Interestingly, the substrates were applied directly on rat wounds that were infected with drug-resistant Gram-positive *Staphylococcus aureus* (DR-*S. aureus*) and Gram-negative *Escherichia coli* (DR-*E. coli*). Results indicated that the Cu-hydrogel + H_2_O_2_ can kill almost all the bacteria in the wound, performing much better than hydrogel alone or H_2_O_2_ alone. This strong antibacterial activity can be imputed to synergic effects between (i) CuNPs that exert their inhibition activity against bacteria according the mechanisms explained before (see Antimicrobial Action of Copper Nanoparticles) and (ii) the transformation of low levels of hydrogen peroxide in ROS that results in high toxicity for drug-resistant bacteria. Moreover, wound healing is evidently improved from a visual inspection and from histological analysis of skin tissue.

### CuNPs in Natural Fibers

A wound dressing can benefit in terms of durability and resistance when a resilient material is used. Fibers, cellulose, and tissues are traditionally used in topical medicaments and wound protection. Exploiting the combination with nanomaterials and other material as fibers could significantly enhance the functionality of such dressings.^77^ Bacterial cellulose is a peculiar polymer with a structure composed of nanofibers of cellulose arranged in a network. The name derives from the fact that it is synthesized by bacteria, which allow high purity and strength without the necessity of refining treatments.^78^ For its biocompatibility, cost-effectiveness, ability to hold/release water, and rheological properties, bacterial cellulose appears to be a good candidate for wound dressing design.^79^ Concerning CuNP composites as antimicrobial materials, nanocellulose is used as a substrate for copper-hydrogel deposition or as a surface to directly grow CuNPs. A polysaccharide-based hydrogel on cellulose with *in situ*-prepared CuNPs (20–50 nm) was recently described by Gutierrez *et al*.^80^ An alginate/bacterial cellulose hydrogel was 3D-printed, adding a reducing agent dropwise and producing incorporated CuNPs. Briefly, alginate beads were prepared by ionic cross-linking and by their incubation with a divalent ion salt. Then, copper nitrate was added and reduced by the dropwise addition of NaBH_4_. This material was finally 3D-printed on bacterial cellulose. The 3D structures demonstrated antimicrobial activity against *E. coli* and *S. aureus* strains *in vitro*. Similarly Chowdhury *et al*.^81^ soaked CuNPs in oil palm empty fruit bunch (EFB), which has no antibacterial activity *per se* but acquires an evident antibacterial and antifungal activity if copper is present. Both *E. coli* and *S. aureus* were killed after 1 h of exposure.

Cellulose nanofibrils oxidized with (2,2,6,6-tetramethylpiperidin-1-yl)oxyl (TEMPO) widen the possible uses of cellulose-based nanocomposites. For the preparation, pulp of Norwegian spruce is subjected to defibrillation by a TEMPO-mediated oxidation and mechanical disintegration.^82^ The material is finally homogenized and poured on a Petri dish to form a 10 μm film. It is demonstrated that this material is particularly prone to adsorption of metal oxides, dyes, and other active species. Valencia *et al*.^82^ grew Cu_2_O NPs on TEMPO-oxidized cellulose nanofibril (TOCNF) exploiting the reduction capabilities of the aldehyde groups present in TOCNF to produce copper in the metallic form. The obtained substrate was able to remove efficiently a dye from an aqueous solution and effectively inhibit Gram-negative (*E. coli*) and Gram-positive (*Listeria innocua*) bacteria.

Another very common material used as substrate for wound dressing that is suitable for incorporating NPs for generating antimicrobial activity is cotton fabric. Its popularity derives from the fact that cotton fabrics possess excellent properties, such as biodegradability, softness, hygroscopicity, and regeneration properties. It is possible to incorporate CuO NPs to generate a bactericidal coating against Gram-negative and Gram-positive pathogens.^83^ Combination with an extract of *Sida acuta* leaves can promote a more efficient activity in killing bacteria against *E. coli*, *P. vulgaris*, and *S. aureus*.^84^

**Figure 5:**
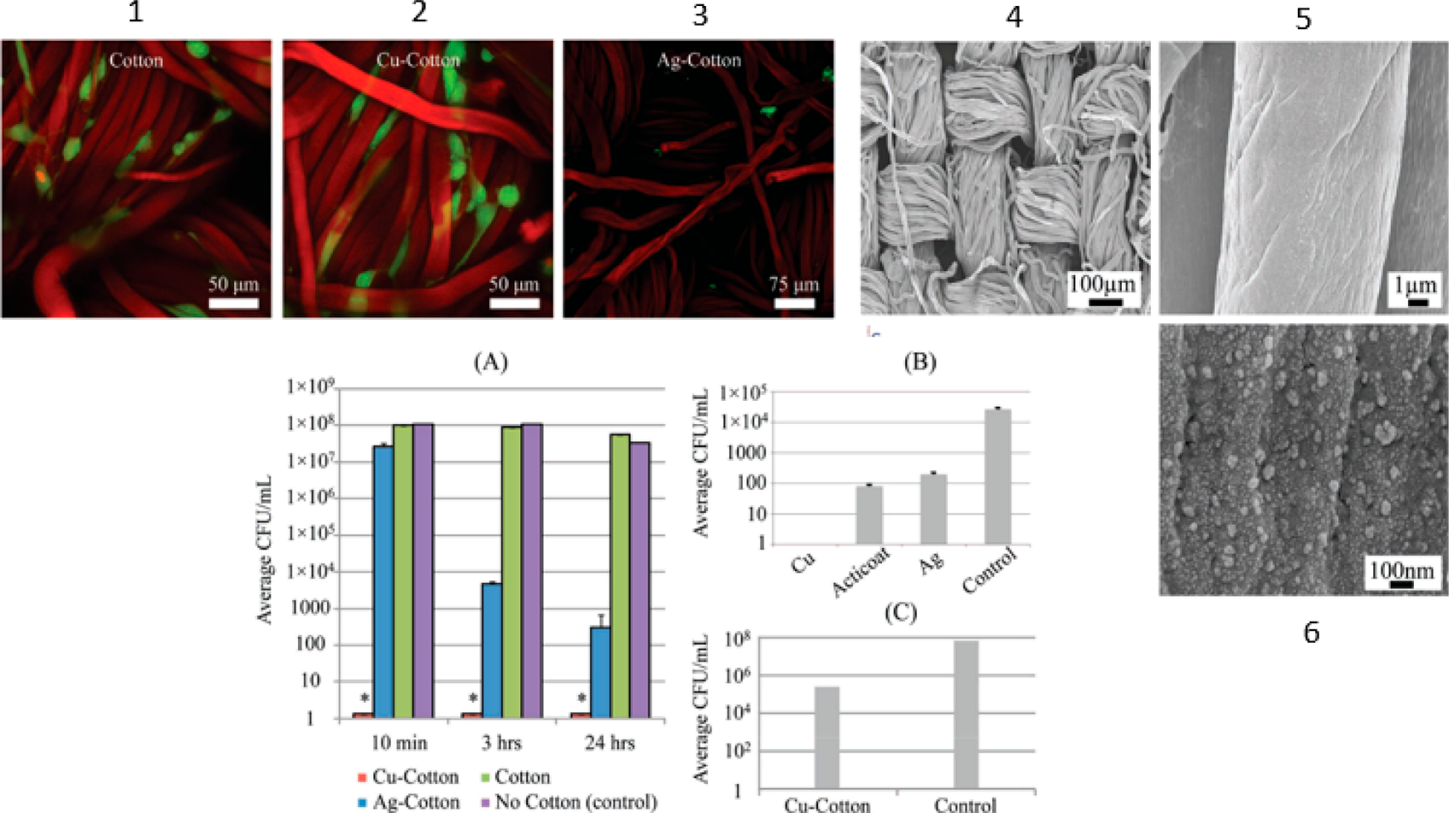
Live–dead staining at laser scanning confocal microscopy are in picture 1, 2, and 3 (red cells = dead, green cells = dead, fibers = red for autofluorescence). SEMs of copper–cotton substrates are in picture 4, 5, and 6. Graph A: Antimicrobial activity against *A. baumannii* at different times. Graph B: A direct comparison among Cu- and Ag-coated cotton substrates and a commercial silver wound dressing, Acticoat. Graph C: Plot showing about 3-log kill for Cu-cotton samples in the presence of *A. baumannii*. Reprinted with permission from ref (77). Copyright 2011 John Wiley and Sons.

Natural cotton fibers can be converted into carboxymethyl cotton fibers with chloroacetic acid for CuNPs grafting.^77^ Briefly, anionic cotton is prepared and immersed in a solution of copper sulfate. The chelated ions are reduced and generate CuNPs. The metal NP-coated cotton substrate gains efficient antimicrobial activity against the MDR bacterium *A. baumannii*. In comparison with just copper ions, the NP-coated cotton substrate is efficient at a total copper concentration that is about 20-fold lower. This was demonstrated by a zone of inhibition assay and a growth inhibition assay. These tests also showed a more pronounced antibacterial activity of the copper substrate in comparison with a commercial one and the analogous silver substrate (Figure 5). These last two substrates are proven to release metal ions in the medium, while only traces of copper ions are detected from the copper substrate in the medium. From a test of compatibility with mammalian cells, copper-coated substrate allows the growth of cells, differently from Ag substrates, probably due to an uptake of copper into these cells.^77^

Marković *et al*.^85^ investigated the release of Cu^2+^ ions from a similar nanocomposite into physiological saline solution when changing the amount of copper. Cotton fibers were treated with (3-aminopropyl)triethoxysilane (APTES) for the complexation reaction with copper and subsequently reduced to NPs. Despite the different Cu content between the samples (from 117 μmol/g to 26 μmol/g), the release of copper ions in the first 9 h was comparable (1.5 μmol/g). Both substrates can effectively inhibit the antibiotics-resistant bacteria *E. coli*, *K. pneumoniae*, and *S. aureus*, while only the sample comprising more copper can ensure the reduction of *C. albicans*. On the other hand, the extract of the more concentrated sample was toxic for skin cells, reducing their viability to 80%.^85^

## CuNPs-Assisted Photothermal Ablation of Microbes

Photothermally active NPs can convert light into heat.^8600,8601^ Under light irradiation with a specific wavelength, these NPs can generate a local and rapid increase of temperature. This approach can be exploited for fast and efficient bacteria eradication, even those resistant to conventional drugs. It could be a great advantage for some specific and precise applications since it can be exogenously triggered on demand. Plasmonic NPs or inorganic, carbon-based materials, chalcogenides, and metal oxide NPs are just some of the materials that can produce photothermal activity. CuSNPs behave like a semiconductor material that strongly absorb in the NIR region (mainly 900–1200 nm) by promoting electrons to the valence band and exerting heat emission by their relaxation.^86,87^ CuSNPs are cheaper than noble metal NPs, and the thermic effect is conveniently combined with the intrinsic chemical antibacterial action generated from the ion release and ROS production.^88^ Moreover, CuNPs combined with photothermal effect are demonstrated to be effective in enhancing tissue regeneration. *In vitro* studies^89^ show that Cu_2_S nanoflowers incorporated in biopolymer fibers exhibit controllable photothermal performance under irradiation, support the spreading and the regeneration of human dermal fibroblasts, and accelerate the migration of endothelial cells. Similar results were shown *in vivo* by Zhou *et al*.,^90^ where they combined the CuS properties with the advantages of hyaluronic acid hydrogel. The increase in temperature achieved upon 808 nm irradiation of different concentrations of CuNPs (10, 20, 50, 100, and 200 μg/mL) went up to 50 degrees above the room temperature. Collagen deposition and regulated expression of VEGF on wounds on rats demonstrated the improvement in wound healing caused by the irradiated CuS-hydrogel with respect to hydrogel alone, irradiation alone, and CuS-hydrogel alone.

Few investigations on the antimicrobial photothermal treatment using CuNPs, CuS NPs, or CuS nanodots, alone or in hydrogels, are reported in the literature. Generally, the photothermal treatment exerts an intense antimicrobial activity, directly dependent on the concentration of the particles and the laser power. In the work of Tao *et al*.,^54^ CuNPs chelated with *N,N*-bis(acryloyl)-cystamine (BACA) were radically polymerized with methacrylate-modified gelatin. A 3D network was generated where the closeness of CuNPs produced a localized surface plasmon with resonance at 808 nm. At this wavelength, the CuNPs-hydrogel can induce a temperature increase by up to 40 degrees in 4 min, depending on the laser power and the copper concentration.

**Figure 6:**
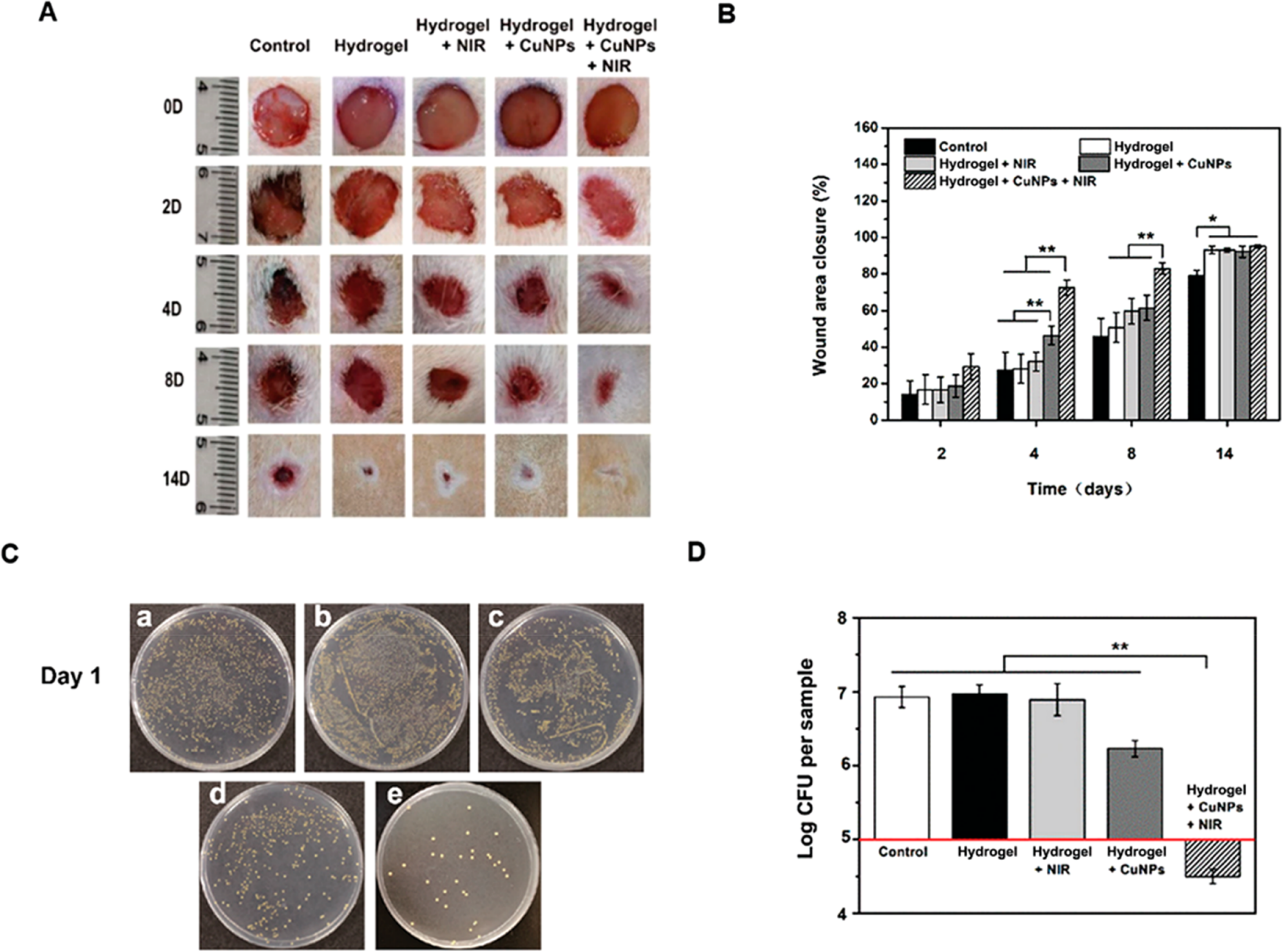
(A) Steps of healing of infected wounds in mice on days 0, 2, 4, 8, and 14. (B) Wound area closure (%) at different times points. (C) Bacteria from the wound tissues on LB agar plates (a: control; b: hydrogel; c: hydrogel + laser; d: Cu-NP-embedded hydrogel; e: Cu-NP-embedded hydrogel + laser). (D) Log of total bacterial CFU on the LB agar plates. Reprinted with permission from ref (54). Copyright 2013 Royal Society of Chemistry.

The CuNPs hydrogel, even with irradiation, is compatible with the proliferation of endothelial cells and does not produce any toxic effect, well mimicking the extracellular matrix. *In vitro* experiments demonstrate the ability of CuNPs hydrogel to kill both Gram-positive (*S. aureus*) and Gram-negative (*E. coli*) bacteria with no irradiation, and even more when irradiation is on. CuNPs hydrogel deposited on rat chronic wounds infected with *S. aureus* and regularly irradiated demonstrated a clear acceleration of the healing, reduced inflammation, and promoted angiogenesis (Figure 6).^54^ Due to the smart design of the material, a photothermal effect from CuNPs has been obtained, despite the usually low thermal emission of CuNPs (metallic or oxides) compared to CuS.

**Figure 7:**
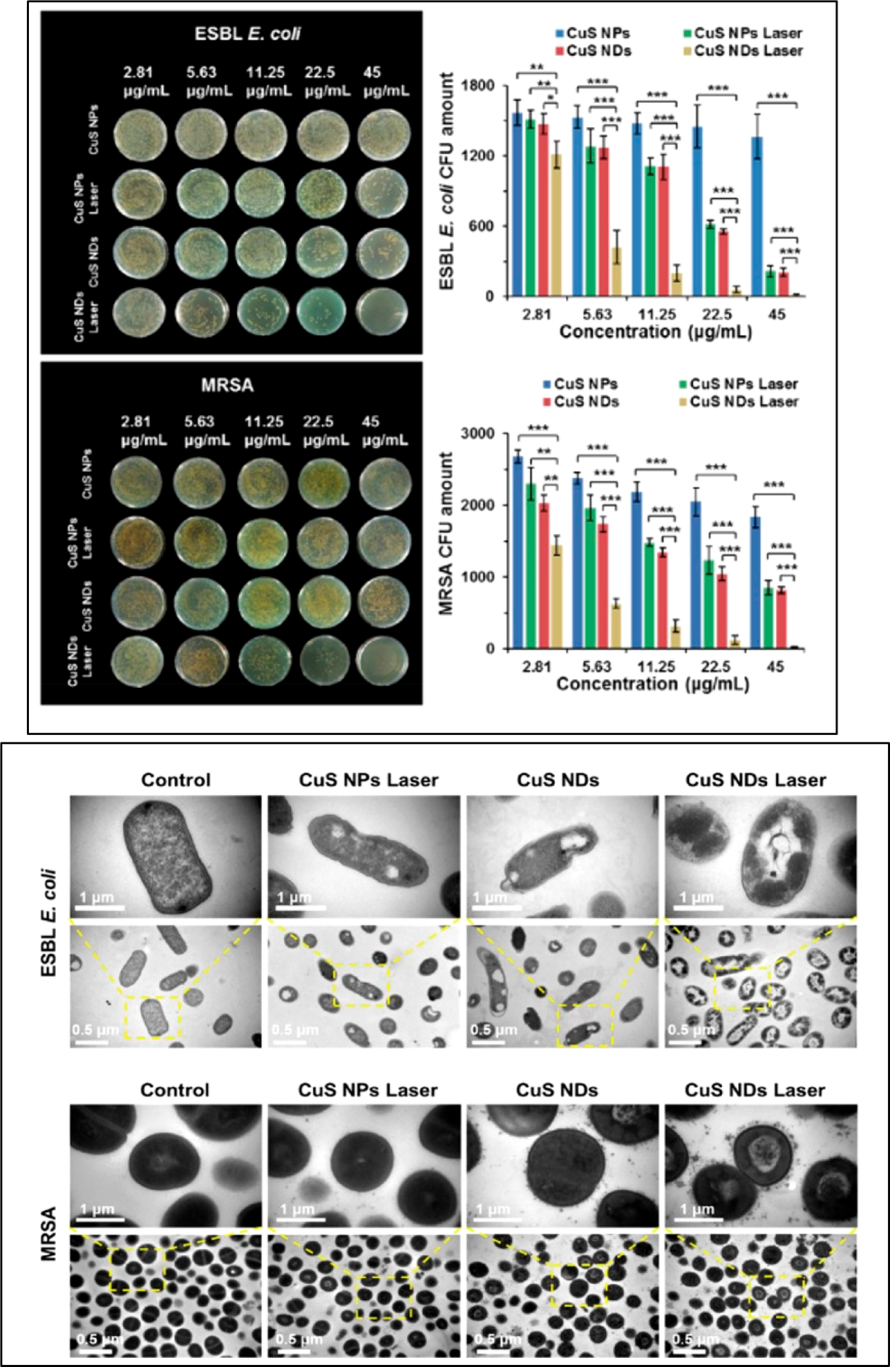
Upper panel: Photos and relative survival rates of ESBL *E. coli* (up) and MRSA *E. coli* (down). In the graphs The amount of bacteria is reported vs the concentration of NPs. Down panel: TEM images of ESBL *E. coli* and MRSA for CuS NPs, CuS NDs with and without laser irradiation (2.5 W/cm^2^, 10 min). Reprinted with permission from ref (91). Copyright 2019 American Chemical Society, with Creative Commons Attribution (CC BY) license.

A paper by Qiao *et al*.^91^ compared the performance in the synergistic antibacterial effect under irradiation of CuSNPs and Cu nanodots (NDs) with and without irradiation. Drug-resistant Gram-negative bacterial ESBL *E. coli* and MRSA were affected *in vitro* from all four situations, but the antimicrobial effects of CuSNDs plus NIR laser irradiation was particularly relevant. Indeed, the researchers detected a higher ROS production for the ultrasmall CuSNDs (∼6 nm) because of the corresponding higher photodynamic conversion effect. Interestingly, the CuSNPs plus irradiation and CuSNDs with no irradiation produced similar effects in both strains (Figure 7). The morphological changes evidenced from TEM images of treated microbes showed damages after CuSNPs plus laser or Cu NDs treatments such as membrane lysis and loss of integrity. The membrane permeability increases with CuSNDs plus laser treatment.

The CuSNPs combined with light treatment are therefore an efficient tool for cell disruption, making them particularly suitable for synergistic treatment with an even wider field of application. The antimicrobial activity generated in the photothermal treatment can coexist with other treatments, generating an effective and multifunctional therapy. Chitosan-coated hollow CuS can be used in a photothermal immunotherapy approach, where they reduce the tumor growth and stimulate the release of tumor antigens.^92^ Yin *et al*.^93^ used CuSNPs for decorating upconverting NPs covered with NaYF_4_:Mn/Yb/Er and Methylene Blue-doped silica to improve the efficiency of energy transfer. They combined photodynamic and photothermic therapy for the treatment of MDR bacteria infection. The antibacterial activity for both *S. aureus* and *E. coli* bacteria under NIR light was assessed *in vitro*, reaching 50% of viability with 150 μg/mL and 20 min of irradiation. Using a quite high concentration of particles (300 mg/mL) and an irradiation of 20 min, the bacteria were completely killed.

## CuNPs as Antiviral Agents

Pharmaceuticals usually need to be specifically designed for a virus in order to prevent infection. In this regard,^94,95^ NPs have the potential to be employed as cost-effective wide-spectrum antiviral agents due to their mechanisms of action.^96^ Gold and silver NPs have been profusely studied in this field, while less is known about CuNPs’ behaviors. Hang *et al*.^97^ suggested a specific virus inhibition of CuNPs by a direct binding with virion surface that blocks the interaction with cell receptors. Otherwise, several authors suggested that the antiviral activity of CuNPs is associated with an antibacterial-like mechanism: “contact killing” (i.e., structure damage), oxidative stress, DNA degradation, and inhibition of activity.^96,98−100^ The CuNPs can directly bind to viruses and damage the capsids by physical or chemical interactions caused by the generation of toxic species.^96,98−100^ The release of copper ions from NPs may support all the mentioned effects, since Cu ions have a direct antiviral activity.^98,101^ The several properties of CuNPs highlighted in *in vitro* and *in vivo* investigations show the importance that this material can have in antiviral coatings and treatment. Copper(II) chloride is demonstrated to be effective in inhibiting dengue virus type-2 at a concentration of 0.13 μg/mL without any toxicity for Vero cells. The activity is imputed to copper ions interacting with the cysteine residues on the surface of the protease.^100^ Most recently, CuNPs attracted attention because of their potential employment in face masks. Copper oxide-impregnated masks filtered >99.85% of human influenza A virus (H1N1) and avian influenza virus (H9N2), and no traces of the viruses were recovered from the copper-treated masks after 30 min.^99^ Inspired by this, Escoffery *et al*.^102^ presented a CuNPs-infused mask for the neutralization of SARS-CoV-2. They produced CuNPs from a dime using hydrogen peroxide, vinegar, and salt, soaked the solution into a cotton fiber mask, and showed the reusability of the substrate. More studies are required in this direction, but the increasing importance of CuNPs should be noted in the health sector. Furthermore, some *in vitro* investigations evidence the various actions that CuNPs can exert on the virus, among them infection inactivation by avoiding the entrance of the virus in cells, disruption of the virus, or inhibition of their replication.

An *in vitro* study demonstrated by microscopy images that Au/CuS core/shell NPs can rapidly inactivate norovirus GI.1 virus-like particles.^19^ Tavakoli and Hashemzadeh^95^ analyzed the effect of CuONPs on Vero cells after they were infected with herpes simplex 1. The inhibition of the infection was confirmed *via* quantitative PCR and Median Tissue Culture Infectious Dose (TCID50). The maximum nontoxic concentration of CuONPs (100 μg/mL) led to about an 83% inhibition rate (similarly to the antiviral activity of acyclovir at 20 μg/mL), demonstrating a good ability to block the proliferation of the infection.

Hang *et al*.^97^ presented a study in which Cu_2_O NPs were evaluated to both terminate and avoid viral infections. This approach can be of particular interest for certain viruses which are persistent and can lead to relevant consequent pathologies. Hepatitis C virus (HCV) is an interesting example. Indeed, HCV is a major public health problem since it becomes chronic in 80% of cases and can lead to hepatocellular carcinoma or hepatic cirrhosis. The current treatments are quite toxic and often can lead to virus resistance. The results showed that Cu_2_O NPs play a role in the binding and the entry of the virus in hepatic human cells. They can inhibit the infection at a concentration of 2 μg/mL, more efficiently than chloroquine and a specific antibody. If applied at different time points (before and after the infection), the antiviral activity changes: Cu_2_O NPs can inhibit the virus only in an early stage; after 2 h they already lose their activity. They probably interact with the virion surface, avoiding interaction with the cell receptor. Interestingly, a study^94^ reported an *in ovo* investigation to assess the employment of CuNPs as antivirals. CuONPs, prepared with fruit extract of *Syzygium alternifolium*, were used against Newcastle Disease Virus (NDV)-infected chicken eggs at 25, 50, and 100 μg/mL of CuO NPs. The presence of the virus was detect by the hemagglutination (HA) test. At a concentration of 100 μg/mL, CuO NPs demonstrated a strong inhibitory activity, preventing the growth of the virus in the allantoic fluid of the eggs. In these conditions, the viability of the egg is 100%, suggesting the potential of the treatment.^94^

## Biodistribution, Toxicity, and Persistence of CuNPs

**Figure 8:**
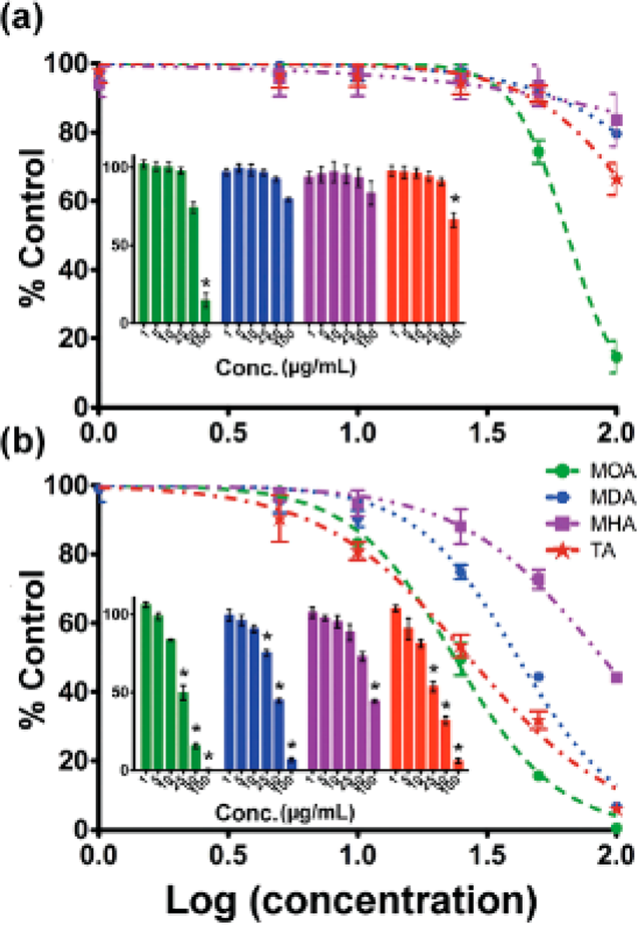
Cells viability normalized on the control vs concentration (logarithmic) of CuNPs at (a) 4 h and (b) 24 h. Lines and colors refer to different surface ligands on NPs (8-mercaptooctanoic acid (MOA), 12-mercaptododecanoic acid (MDA), and 16-mercaptohexa-decanoic acid (MHA)). Reprinted with permission from ref (103). Copyright 2009 Royal Society of Chemistry.

Most of the investigations on CuNPs consider their impact on cell viability by analyzing a range of concentrations of copper in order to elucidate their potential toxicity profile. In general, in addition to the amount of copper, the matrix or the molecules surrounding the NP contribute positively or negatively to the cytotoxicity. An *in vitro* study on the dissolution kinetics of CuNPs showed that the surface functionalization of CuNPs defined the rate of ion release due to modulated oxidation,^103^ affecting their cytotoxicity on human type II alveolar epithelial cells. PEG-CuNPs dissolve faster than other functionalized with thiolated acids. The dissolution rate decreases with the increasing concentration of CuNPs (hypothetically because of the agglomeration of particles), and the maximum dissolution amount is reached in about 8 h.^103^ Almost 100% of the cells remained alive after 4 h from the treatment for concentrations up to 50 μg/mL, while at 100 μg/mL a significant reduction was recorded (Figure 8). After 24 h, a decrease in viability was evident, depending on the increased concentration of copper.

CuNPs in bioactive glasses had no significant toxicity toward fibroblast cells and human mesenchymal stem cells when applied *in vitro* at concentrations of 100, 10, and 1 μg/mL.^104^ HUVEC viability *in vitro* was similar in the presence of CuNPs prepared with *A. saralicum* extract^63^ or *F. vulgaris* extract.^52^ It was above 80% even up to 1000 μg/mL and definitely higher compared to copper sulfate, suggesting the importance of the nanomaterial composition. Similar results were obtained with cashew gum-stabilized CuNPs on murine macrophages and murine fibroblast cells^45^ and with CuS hydrogels after 48 h on mouse embryonic fibroblasts, where the proliferation results were even more promoted.^90^

A prior evaluation of the impact of CuNPs on the functionality of organs and the description of their biokinetics is pivotal for the translation of nanomaterials to the clinical practice.^105−10900,10901^ The information present in the currently available literature does not describe a complete assessment regarding the persistence and accumulation of CuNPs in the body. On the other hand, CuNPs have some advantages with respect to other non-biodegradable metals (i.e., gold). Indeed, they should easily dissolve in biological fluids and be metabolized or excreted. It should be noted that a comprehensive knowledge of the overall clearance process of CuNPs is more difficult with respect to other metals because of the natural presence of copper in living organisms.

**Figure 9:**
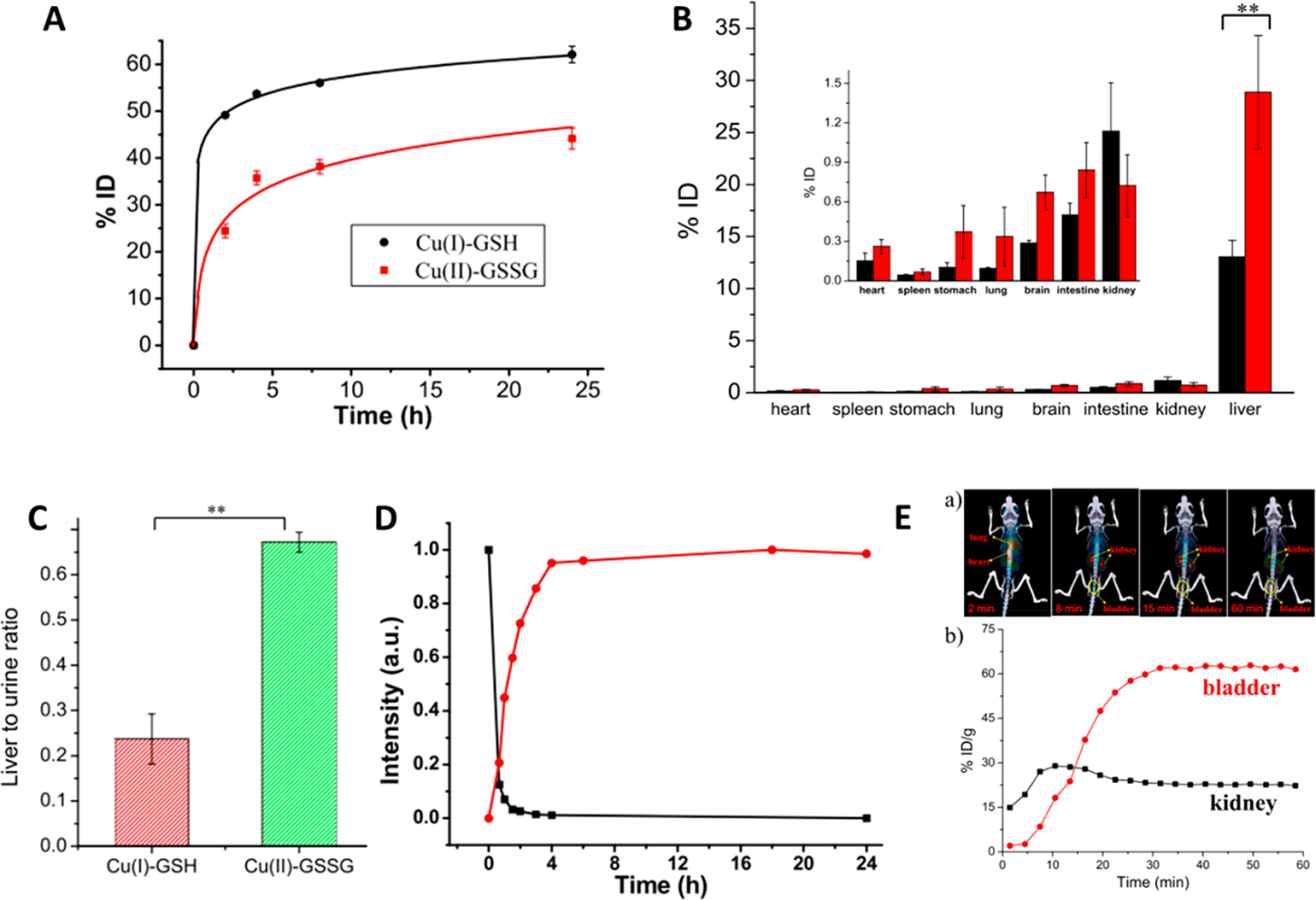
(A) Copper in the urine of mice at different times post injection. (B) Biodistribution of Cu(I)-GSH and Cu(I) complex (Cu(II)-GSSG). (C) Liver to urine ratios of the two complexes at 24 h. (D) Fluorescence intensity of the complexes (black is Cu(I) complex, red the Cu(II) complex). (E) Time dependence of copper distribution in kidneys and bladder. Reprinted with permission from ref (109). Copyright 2017 The Authors, published open access by MDPI under Creative Commons Attribution (CC BY) license.

Yin *et al*. followed the accumulation and clearance in mice of copper ions in different oxidation states after injection of glutathione complex with Cu(I) and the oxidized glutathione disulfide complex with Cu(II)^109^ (Figure 9). The complexes are excreted mostly from the body, with more efficient renal clearance for the first one. Most of the complexes that are not expelled *via* urine and feces accumulates in the liver, 13% ID and 27.5% ID, respectively. The difference in accumulation can be attributed to the valence of the ions, suggesting that Cu(II) complex forms strong bounds with protein and remains in the liver more than Cu(I) complex. Furthermore, fluorescence measurements on *in vitro* samples demonstrate that the Cu(I) complex is fast oxidized to Cu(II), in the first 4 h. Introducing radioactive copper in Cu(I) complex, positron emission tomography on treated mice shows that the metal moves to kidney and then to the bladder rapidly in the first 30 min. It persists mostly in the bladder for the first hours, confirming the rapid clearing of the metal complex to urine.

Yang *et al*.^110^ compared the renal clearance and degradation of 2 nm Glutathion-CuNPs and their released products (Cu(II) complex) after injection in mice. Approximately, the 78.5% ID of Glutathion-CuNPs is excreted through the urine in the first 24 h while only the 22% ID of the Cu(II)complexes is found in the urine. Free ions of copper are demonstrated to bind with caeruloplasmin and hephaestin, remaining easily trapped in the liver. Glutathione reduces this affinity because it creates a zwitterionic shield that influences the charges interaction. Using radioactively marked glutathione-CuNPs, it is demonstrated that they do not bind to serum proteins but they undergo to a gradual dissociation in biological fluids, producing Cu(II)complexes, which partially bind to serum protein. This means that the initial distribution of copper in tissues is due to Glutathione-CuNPs, and in the following 24 h it is strongly influenced by the formation of the ion complex. After 24 h about 30% ID of Cu(II) complex is in the liver and up to 0.9% ID is present in kidneys, lowering to 0.6 in brain and 0.3 in lungs.^110^

**Figure 10:**
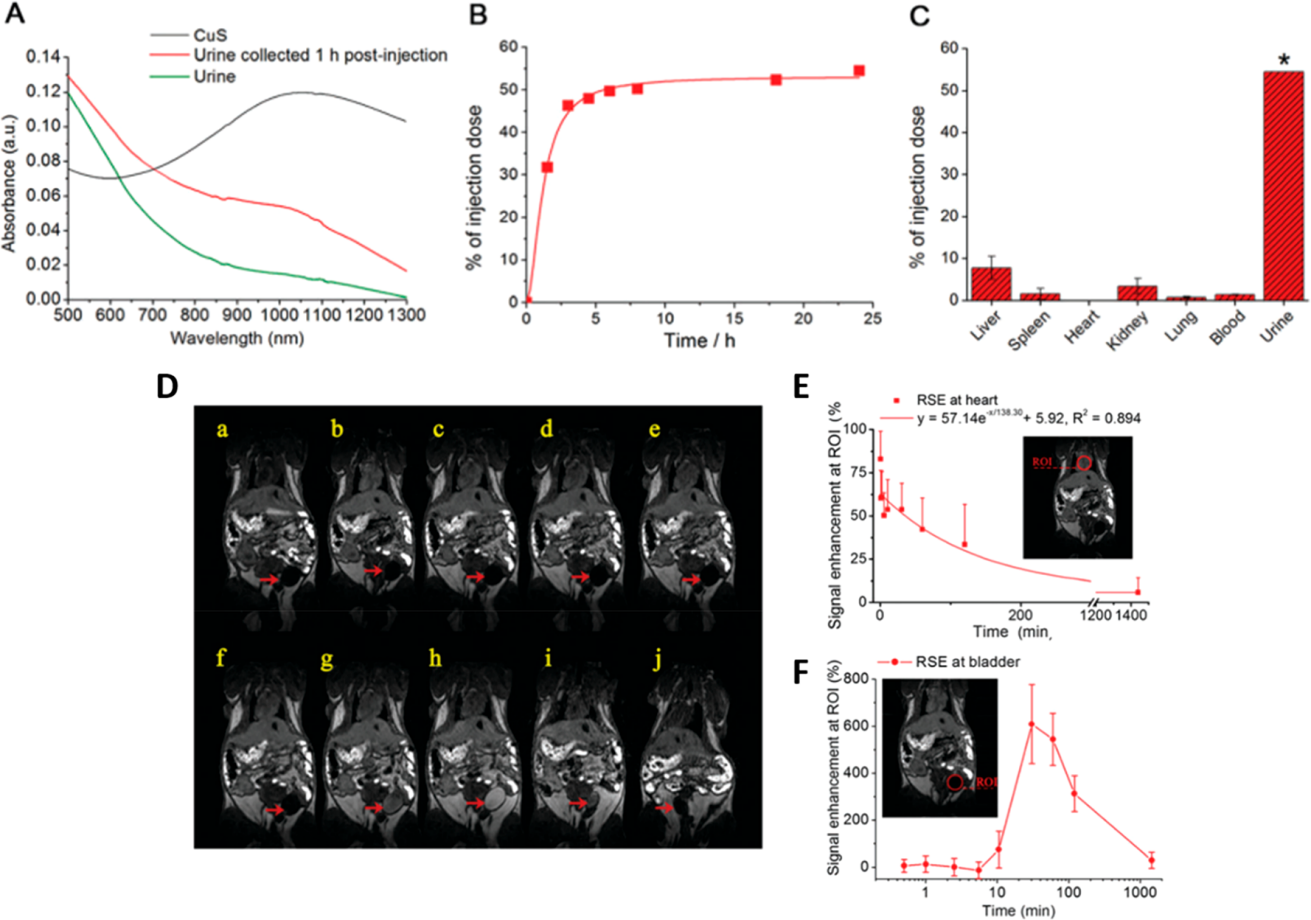
Upper panels: Renal clearance and biodistribution studies of GSH-CuS NDs. (A) Absorption spectra of urine samples before and 1 h after the injection. (B) Amount of Cu excreted in urine. (C) Biodistribution of GSH-CuS NDs at 24 h post-injection. D) MR images of GSH-CuS NDs enhanced (from a to j: pre-injection and 30 s, 1 min, 2.5 min, 5.5 min, 10.5 min, 30.5 min, 1 h, 2 h, and 24 h post-injection signal enhancement in heart (B) and bladder (C) caused by GSH-CuS NDs. Reprinted with permission from ref (111). Copyright 2013 Royal Society of Chemistry.

Liang *et al*.^111^ investigated the renal clearance of glutathione-CuNPs in mice after intravenous injection of 1.8 μg/mL. Specifically, glutathione CuS nanodots (GSH-CuS NDs) are directly revealed in urine samples exploiting their intrinsic NIR absorption (700–1300 nm). In accordance with previous studies, GSH-CuS NDs are found in urine 1 h after the injection reaching the 54.6% ID at 24 h after the administration (Figure 10). The possibility to record their presence in urine by NIR suggest that they are expelled intact.

**Figure 11:**
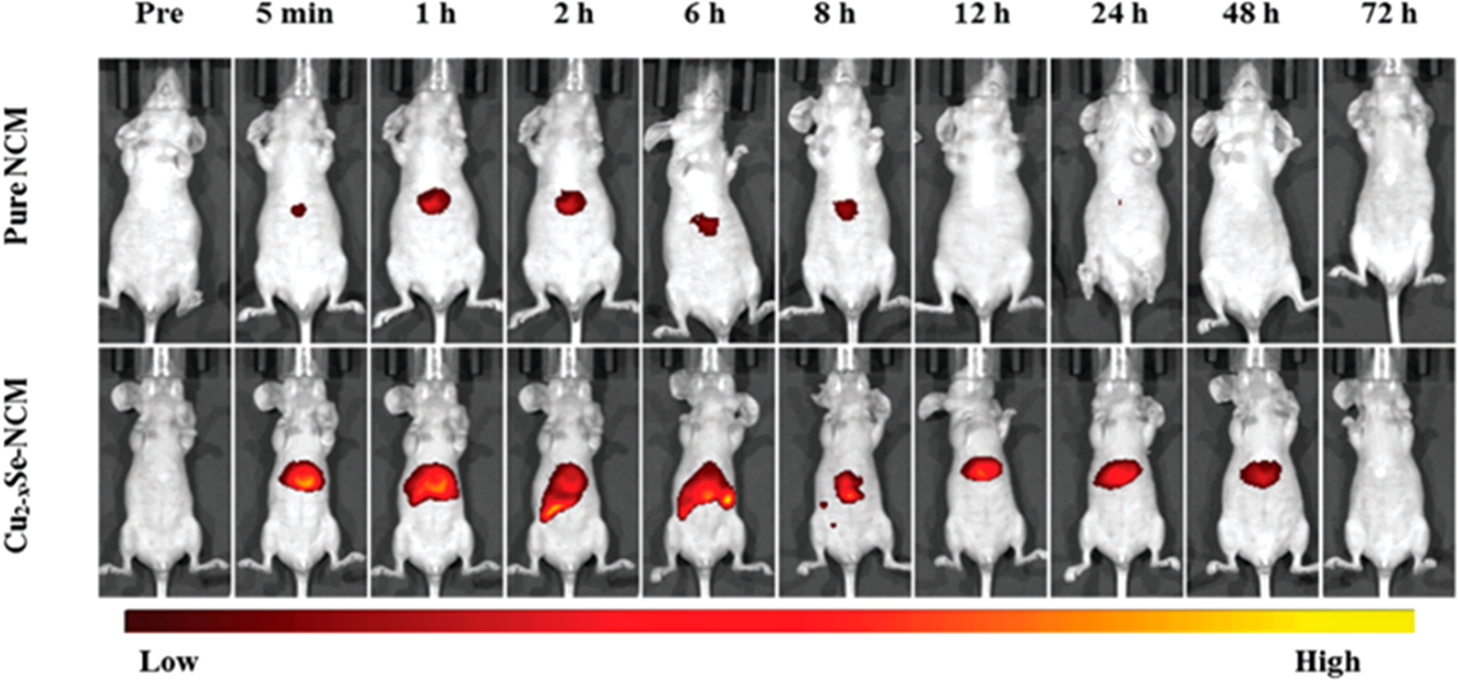
Fluorescence images *in vivo* of mice treat before and after treatment with NCM (400 μM, 200 μL) and Cu_2–*x*_Se–NCM NPs.^114^ Reprinted with permission from ref (114). Copyright 2019 Royal Society of Chemistry.

The *in vivo* distribution of GSH-CuS NDs is furthermore studied by magnetic resonance imaging, taking advantage of the magnetic properties of copper. After injection, a signal enhancement is detected in the heart, slowly disappearing after 2 h, meaning that in that range of time, GSH-CuS NDs can freely circulate in the vessels without being catch by the reticuloendothelial system. At the same time, the signal intensifies gradually in the bladder region, demonstrating that GSH-CuS NDs are filtrated by the kidneys. After 24 h only the 7.7% ID and 3.3% ID accumulate in liver and kidneys, respectively, which is significantly lower compared to the other works mentioned. This result could be attributed to the smaller size of NPs, suggesting that the ultrasmall NPs undergo to rapid renal clearance, faster than bigger NPs, reducing the exposure of the organs to dissociated copper ions. Consistently, to other metals like gold, it is known that the renal clearance efficiencies depends on size, together with shape, charge, and composition.^112^ The supposed threshold for renal clearance of intact metal-based NPs is around 6 nm.^113^ The work of Han *et al*.^114^ confirmed this concept, presenting the distribution and metabolism of ultrasmall copper selenide NPs (Cu_2–*x*_Se). The 2 nm NPs, after being injected in mice intravenously at a concentration of 10 mg/kg, are eliminated mostly in urine and feces in 6–12 h (29.2% and 36.7%, respectively). In the first 2 h, Cu_2–*x*_Se are found located mostly in kidneys (12.1 μg/g of tissue), decreasing then at 72 h (2 μg/g of tissue), confirming the ability of this particles to pass through kidneys and to be excreted rapidly by the urinary system. Concerning the other organs, the lungs accumulation is similar to the kidney one, followed by the spleen and the heart. Liver is an exception, in which the copper content reaches the maximum of 20.8 μg/g of tissue after 4 h from the injection. Interestingly, the copper released was measured *in vivo* in a range of time, thanks to a fluorescent probe specifically designed to activate in the presence of Cu^2+^. After accumulation in the liver, fluorescence diffuses to intestines and spleen, for becoming weaker after 8 h in the liver and more intense in the intestines. The fluorescence disappear after 72 h, demonstrating that after releasing copper in the liver, it is metabolized to intestines and totally eliminated with feces after 72 h (Figure 11).^114^

In order to elucidate the influence of the copper release to organs in mice, the authors characterize several proteins in the liver that are involved in copper storage and transport. As instance, ATP7B protein usually acts for copper trafficking and export of copper in excess out of the cell, regulating the copper content in the bile. Its activity is increased at 12 and 48 h after the injection and became normal again at 72 h. This evidence is consistent with degradation of Cu_2–*x*_Se NPs and with a consequent increase of ion that makes the liver cell decrease the uptake and increase the elimination of copper ions. Importantly, the histological analysis on major organs do not show any cell damage at day 1, 3, 10, 20, and 28 (dose: 10 mg/kg), highlighting the very low toxicity of the treatment.

No damage in organs is found as well if 120 nm CuS nanoplates are injected in mice at a concentration of 5.5 mg/kg.^115^ In this work, the concentration of copper in organs is measured *via* inductively coupled plasma atomic emission spectrometer and subtracted to the normal content of copper in the organs. The level in liver and kidneys increases to 1.6 and 0.9 μg/g at 4 h after injection and decreases again at 48 h. Lungs and liver follow a comparable trend while the spleen reaches 2.4 μg/g after 30 min, to decrease rapidly after. Overall the findings are similar to what described in the work of Han *et al*.:^114^ most of the copper is rapidly excreted *via* urinary system and feces with no toxic effect on organs after injection. Considering the acute toxicity, the maximum tolerated dose is calculated to be 8.66 mg/kg and the medial lethal dose is 54.5 mg/kg.

Dey *et al*.^116^ determined the biodistribution of about 50 nm copper oxide NPs, comparing two different synthetic ways: chemical and green with *Azadirachta indica*. They demonstrated that even for lower doses of CuNPs (1–0.1 mg/kg), the accumulation in liver and spleen is significant and increases by increasing the injected amount of CuONPs. Interestingly, almost no toxicological effect was noticed only with the mice treated with the green NPs from the histopathological images on organs after 15 days of treatments. This confirms that the conjugation of the NPs is a relevant factor for modulating the toxicity. Hepatotoxicity is also found on mice orally treated with up to 6.5 mg/kg of 40–60 nm CuNPs in a diet of 4 weeks.^117^ Compared with a copper carbonate diet, CuNPs are better adsorbed in the digestive tract than copper from the other source, in accordance with a lower copper excretion in feces and urine. These results may suggest that a long-lasting treatment with CuNPs orally administered can expose the digestive system to a stress that can bring to relevant tissue damage. Lei *et al*.^118^ also detected liver damage in mice orally treated with a shorter regime but with a higher dose of NPs (5 days, 200 mg kg^–1^ d^–1^ of CuNPs). Furthermore, Cholewinska *et al*.^117^ noticed a significant accumulation of copper in the brain of mice after a 4 weeks CuNPs diet, higher with respect to the carbonate-based diet. The CuNPs are, hence, able to move from the digestive system to blood, cross the blood–brain barrier, and significantly accumulate in organs. The potential adverse effects of CuNPs accumulation in rat brain were recently studied by Fahmy *et al*.^119^ They monitored specific oxidative stress parameters and acetylcholinesterase (AChE) activity after intravenously injecting rats for 2 days with 15 mg/kg of 14 nm CuNPs. The quantification of copper in the different areas of the brain reveals an increase in all brain areas, except the medulla and mid brain. Hippocampus contains 0.04 mg of copper per grams of tissue, which is the highest value among all areas. Overall, significant changes in oxidative stress are detected together with inflammation and changes in the behavior of rats (decrease in exploring motion up to 54%).^119^ These results are coherent with a precedent work^120^ where, alternatively to intravenous injection or oral exposure, CuNPs are administered *via* intranasal doses. A few microliters of 25 nm CuNPs at concentrations up to 40 mg/kg are instilled in the naris of the mice three times over a week. In addition to oxidative stress, CuNPs seems to cause lesions in olfactory cells in hippocampus and influences the nervous system.^121^ If the dose is reduced to 1 mg/kg, no lesion is observed, neither in the brain or liver.^122^

The effect of a long-term exposure to copper as ion generates a not negligible adverse effects in the brain. A study from Pal *et al*.^123^ described long-term copper toxic effects in rats after injection of copper lactate solution (0.15 mg Cu/100 g BW), daily for the period of 90 days. They observed brain swelling and increased number of astrocytes, accompanied by impaired spatial memory and neuromuscular coordination. Accordingly, Kardos *et al*.^124^ found that the free excess of copper accumulates close to the cerebral cortex and hippocampus. It is supposed that a long-term exposure to an excess of copper ions can be caused by a high fat diet.^25^ Concerning the long-term effects of an excess of copper ion in the body, it is important to notice that only a few works have investigated CuNPs. Copper ions are reported from several studies to have an impact in the immune system, in ovaries, and in nervous system.

Relevant is the involvement of copper in the development of Alzheimer’s disease. The metal has high affinity for the β-amyloid peptide, altering the structure, enhancing the agglomeration and formation of plaques, which are the cause of the disease.^25^ Not only copper as ion has this effect, but also environmental CuONPs can be associated with the promotion of Alzheimer’s disease.^125^ In a recent *in vitro* study, two human cell lines (human neuroblastoma and human neuroglioma) have been incubated for 1 day with a quite high concentration of CuONPs (100 μM). By monitoring the pathological features connected with Alzheimer’s disease, an increase in primary components of β-amyloid plaques, probably due to oxidative stress, has been observed. This suggests the possible implication of CuONPs in the development of Alzheimer’s disease, even if, at the moment, *in vivo* confirmations are still missing. Zhang *et al*.^126^ recently investigated *in vivo* Fe_*x*_Cu_*y*_Se NPs for the treatment of Alzheimer’s disease. The NPs, functionalized with penicillamine, show a promising ability of inhibition of β-amyloid 42-monomer aggregation and of the disaggregation of β-amyloid fibrils.^126^

Inhalation of CuNPs is reported to cause lung inflammation, increasing the level of total proteins in bronchoalveolar fluids.^127^ Furthermore, after 1 week of exposure to 5 mg/kg, histopathological analysis revealed a certain degeneration of lungs, fibrosis, and granuloma.^128^ When 25 nm CuNPs were coated with chitosan and administered *via* nasal instillation and about 30 μg of copper was deposited per mouse (*via* tracheal incision), the inflammation was increased with respect to treatment with CuNPs without chitosan. The polysaccharide is responsible of this effect since it has severe proinflammatory effects on lung tissues^129^ and it adheres well to the mucus, prolonging the exposure to metal and avoiding the clearance.^130^

The biodistribution, toxicity, and persistence investigations on CuNPs are summarized in Table 2.

**Table 2:** Summary of Biodistribution, Toxicity, and Persistence Investigations on CuNPs

authors	toxicity and persistence	administration
Shi *et al*., 2017^103^	100% of human type II alveolar epithelial cells were alive 4 h after the treatment for concentrations up to 50 μg/mL, while at 100 μg/mL a significant reduction was recorded.	*in vitro*
Zheng *et al*., 2017^104^	Viability was above 80% up to 1000 μg/mL for human mesenchymal stem cells.	*in vitro*
Amorim *et al*., 2019^45^	Cashew gum-stabilized CuNPs on murine macrophages and murine fibroblast cells, above 80% even up to 1000 μg/mL.	*in vitro*
Zhou *et al*., 2020^90^	CuS hydrogels after 48 h on mouse embryonic fibroblasts, above 80% even up to 1000 μg/mL.	*in vitro*
Yin *et al*., 2017^109^	GSH-CuNPs persisted mostly in the bladder for the first hours, with rapid clearing of the metal complex to urine.	injection
Yang *et al*., 2015^110^	78.5% ID of glutathione-CuNPs was excreted through the urine in the first 24 h, and 22% ID of the Cu(II)complexes was found in the urine. After 24 h, about 30% ID of the Cu(II)complex was in the liver and up to 0.9% ID was present in the kidneys, lowering to 0.6% ID in brain and 0.3% ID in lungs.	injection
Liang *et al*., 2017^111^	After 24 h, only 7.7% ID and 3.3% ID accumulated in liver and kidneys, respectively; also detected in the heart, slowly disappearing after 2 h.	injection
Han *et al*., 2019^114^	Ultrasmall copper selenide NPs, 10 mg/kg, are eliminated mostly in urine and feces in 6–12 h (29.2% and 36.7%, respectively). In the first 2 h, Cu_2–*x*_Se was found located mostly in the kidneys (12.1 μg/g of tissue), then decreased at 72 h (2 μg/g of tissue).	injection
Feng *et al*., 2015^115^	120 nm CuS nanoplates at a concentration of 5.5 mg/kg; levels in liver and kidneys increased to 1.6 and 0.9 μg/g at 4 h.	injection
Dey *et al*., 2019^116^	Hepatotoxicity was also found on mice orally treated with up to 6.5 mg/kg of 40–60 nm CuNPs in a diet for 4 weeks.	orally
Lei *et al*., 2015^118^	Liver damage in mice orally treated with a shorter regime but with a higher dose of NPs (5 days, 200 mg kg^–1^ d^–1^ of CuNPs).	orally
Cholewińska *et al*., 2018^117^	Accumulation of copper in the brain of mice after a 4 weeks of CuNPs diet; higher with respect to the carbonate-based diet.	orally
Fahmy *et al*., 2020^119^	Accumulation in all brain areas, except the medulla and mid brain; hippocampus contained 0.04 mg of copper per gram of tissue.	intranasal
Shi *et al*., 2020^125^	Increase in primary components of β-amyloid plaques *in vitro*.	*in vitro*
Sandhya Rani *et al*., 2013^128^	After inhalation, a certain degeneration of lungs, fibrosis, and granuloma.	inhalation

## Conclusions and Perspectives

In summary, copper NPs are effectively promising materials in the management of infectious and communicable diseases. The antimicrobial activity of copper is widely efficient on a plethora of microorganisms, including several multi-drug-resistant ones. CuNPs can provide a joint action against pathogens that combines penetration and disruption of the microbes’ structure (governed by the shape and the size of the NPs), the intense ROS generation by CuNPs, and the generation of superoxide molecules from released copper ions. With respect to gold and silver NPs, CuNPs demonstrated comparable or enhanced antimicrobial activity associated with a lower cost (copper is about 8 time less expensive than silver) and an increased biocompatibility. Indeed, CuNPs generate copper ions in the surroundings that contribute to the normal healthy processes of the human tissues. Copper is a cofactor for metalloproteins and enzymes, and it is essential to aerobic forms of life. It can enhance the regeneration of the tissues by increasing the quality of the skin. Consequently, this evidence suggests that CuNPs are probably the best candidates for the development of the next technologies for management of infectious and communicable diseases, where these synergistic mechanisms can be of great advantage. Further studies are needed to shed light on the antimicrobial mechanism of different CuNPs in terms of chemical composition, size, shape, and biological identity. This would help to maximize the antimicrobial effect as well as to increase the health of the tissues and minimize the toxicity. Moreover, the local toxicity and the long-term effects certainly need to be considered. It should be noted that pharmacokinetics and pharmacology information for “absorption, distribution, metabolism, and excretion” (ADME) of CuNPs in living organisms have still to be comprehensively investigated together with the biosafety profiles in different vertebrate models. A few recent investigations have demonstrated that the administration pathway of CuNPs (oral, injection, tracheal deposition) dramatically influences the fate of the nanomaterial. In general, consistent and wide data are missing concerning biokinetics and biodistribution, and even less is known about long-term persistence in the body and the environment. A rational design of nanocomposites and nanostructured materials based on copper is expected to gain much more importance in the future in a variety of applications, including antimicrobial tissues and surfaces, topical treatment, antivirals, and oncology. The works reported in this Review highlight that copper exerts toxicity only if present in excess with respect to the amount that can be metabolized from the organism. For this reason, a strong control over the amount of copper injected/released is pivotal to minimize the adverse effects. Thus, slow-releasing NPs can be crucial to ensure a suitable level of copper for efficient antimicrobial activity while avoiding an overload. In this regard, core–shell NPs such as the passion fruit-like nano-architectures are of particular interest because they comprise ultra-small NPs in a biodegradable silica nanocapsule that can be modulated to control the metal release.^106,108,13100,13101^ The toxicity of CuNPs can be also regulated by exploiting their interaction with the environment. The surface ligands of NPs and the interplay with environmental protein (protein corona) can influence the uptake in cells and the interactions with organs, giving a relevant effect in the metabolic pathways, persistence, and toxicity.^131−133^ Regarding CuNPs and protein corona formation, very few works can be found. Recently, Akhuli *et al*.^134^ investigated the interactions between bovine serum albumin and copper nanoclusters modified with tannic acid, chitosan, or cysteine, reporting that CuNPs interact with BSA without modifying its structure. Investigations on bio/nano interactions and protein corona formation are of particular interest in order to shed light on ADME and the cellular uptake. In summary, in order to unleash the full potential of CuNPs, more comprehensive investigations on their rational design as well as on their interactions with organisms are urgently demanded.

## Vocabulary

**copper-based nanoparticles (CuNPs)**: nanoparticles with a diameter between 1 and 100 nm constituted of copper or a copper-based compound
**reactive oxygen species (ROS)**: free oxygen radicals that may cause damage to DNA, RNA, and proteins, and may cause cell and microbe death
**vascular endothelial growth factor (VEGF)**: signal protein produced by cells for stimulating the process of formation of blood vessels
**human umbilical vein endothelial cells (HUVEC)**: cells derived from the endothelium of veins from the umbilical cord
**absorption/distribution/metabolism/excretion/toxicity (ADMET)**: a set of criteria to evaluate the behaviors of a therapeutics within an organism
